# Identification of ALDH3A2 as a novel prognostic biomarker in gastric adenocarcinoma using integrated bioinformatics analysis

**DOI:** 10.1186/s12885-020-07493-x

**Published:** 2020-11-04

**Authors:** Zhenhua Yin, Dejun Wu, Jianping Shi, Xiyi Wei, Nuyun Jin, Xiaolan Lu, Xiaohan Ren

**Affiliations:** 1 Department of Digestive, Shanghai Pudong Hospital, Fudan University Pudong Medical Center, 2800 Gongwei Road, Shanghai, 201399 China; 2 Department of General Surgery, Shanghai Pudong Hospital, Fudan University Pudong Medical Center, 2800 Gongwei Road, Shanghai, 201399 China; 3 The State Key Lab of Reproductive, Department of Urology, The First Affiliated Hospital of Nanjing Medical University, Nanjing, 210029 China

**Keywords:** Bioinformatics analysis, Prognosis, ALDH3A2, Immune cells

## Abstract

**Background:**

Extensive research has revealed that genes play a pivotal role in tumor development and growth. However, the underlying involvement of gene expression in gastric carcinoma (GC) remains to be investigated further.

**Methods:**

In this study, we identified overlapping differentially expressed genes (DEGs) by comparing tumor tissue with adjacent normal tissue using the Gene Expression Omnibus (GEO) and the Cancer Genome Atlas (TCGA) database.

**Results:**

Our analysis identified 79 up-regulated and ten down-regulated genes. Functional enrichment analysis and prognosis analysis were conducted on the identified genes, and the fatty aldehyde dehydrogenase (FALDH) gene, ALDH3A2, was chosen for more detailed analysis. We performed Gene Set Enrichment Analysis (GSEA) and immunocorrelation analysis (infiltration, copy number alterations, and checkpoints) to elucidate the mechanisms of action of ALDH3A2 in depth. The immunohistochemical (IHC) result based on 140 paraffin-embedded human GC samples indicated that ALDH3A2 was over-expressed in low-grade GC cases and the OS of patients with low expression of ALDH3A2 was significantly shorter than those with high ALDH3A2 expression. In vitro results indicated that the expression of ALDH3A2 was negatively correlated with PDCD1, PDCD1LG2, and CTLA-4.

**Conclusion:**

We conclude that ALDH3A2 might be useful as a potential reference value for the relief and immunotherapy of GC, and also as an independent predictive marker for the prognosis of GC.

## Background

GC arises from the epithelial cells of the gastric mucosa and is a common malignancy of the digestive system that results in an estimated 990,000 new cases and 738,000 deaths each year [1]. The onset of GC can be seen at any age but is typically more common in men with an approximately 2:1 male-to-female ratio [1]. Approximately 90% of GC are adenocarcinomas (STAD; stomach adenocarcinomas), of which the two most frequent histological subtypes are classified as well-differentiated (or intestinal type) and undifferentiated (or diffuse type). GC is known to be a multifactorial disease, and the incidence of GC is associated with a range of factors, including helicobacter pylori infection, dietary factors, tobacco, obesity, and others [2]. The incidence and mortality of GC have dropped dramatically in the past few decades due to increases in early screening and planned prevention [3]. However, despite the reduction in incidence and mortality, GC remains a serious public health problem worldwide.

Presently, the combined application of multiple treatments, including surgery, chemotherapy, radiotherapy, and targeted gene therapy, has markedly improved the survival rate of GC. Unfortunately, the overall 5-year relative survival rate is still below 20% worldwide, except in Japan [4]. Increasingly, the investigation of GC has been focused at the molecular level. For example, studies from Wong et al. and Sawaoka H et al. indicated that COX2, a membrane conjugated protein, might play a pivotal role in cell proliferation, apoptosis, angiogenesis, and gastric carcinogenesis [5, 6]. Vascular endothelial growth factor (VEGF), matrix metalloproteinase (MMP)-2, and MMP-9 also were found to be related to the metastasis of GC [7]. Moreover, according to recent studies, the abnormal expression of non-coding RNAs, especially microRNAs (miRNAs) and long non-coding RNAs (LncRNAs), target specific mRNAs to form a complex regulatory network that influences gene expression [8, 9]. Therefore, it is critical to identify useful biomarkers that can be used for the early diagnosis and prognosis of GC.

Recently, the rapidly developing, high-throughput platforms for gene expression have been widely applied for molecular classification, prognosis prediction, and targeting new drug discovery [10]. The broad discipline of bioinformatics can be applied to capture, store, analyze, and interpret biological data utilizing specific algorithms and software. This wide range of functions provided by bioinformatics allows us to identify DEGs of interest as well as functional pathways that are correlated with the occurrence and development of carcinomas. We conducted a series of bioinformatics analyses based on mRNA data obtained from the GEO and TCGA databases to investigate the molecular mechanisms that underly GC. The essential genes we identified were found to be directly associated with the prognosis of GC and are likely candidate biomarkers for GC.

## Methods

### Gene expression profile data

**Fig. 1:**
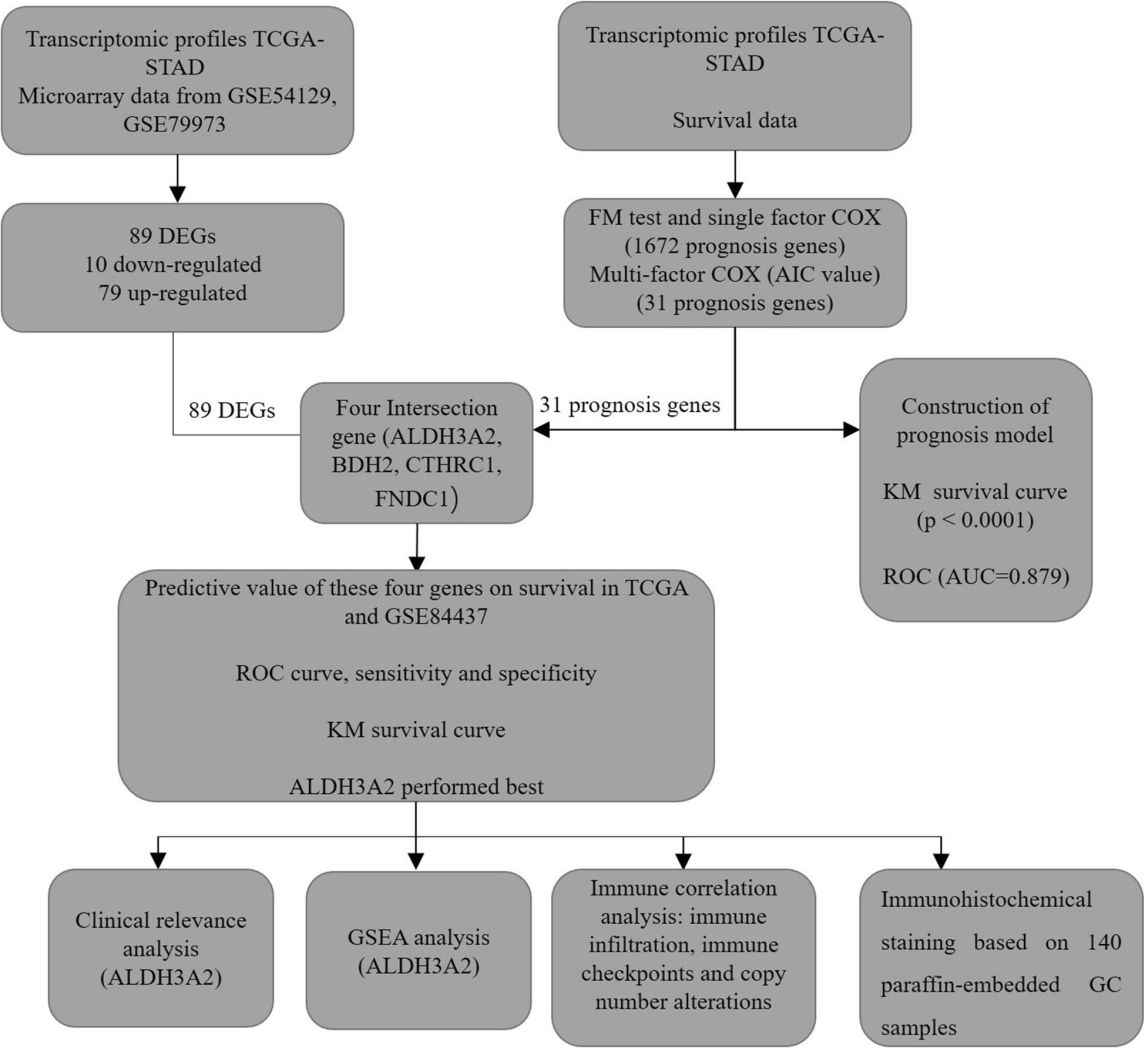
The flowchart of our bioinformatics analysis. Abbreviations: TCGA, The Cancer Genome Atlas; GEO, Gene Expression Omnibus; DEGs, Differentially expressed genes

Two independent GC gene expression profiles, GSE54129 and GSE79973, consisting of 121 primary gastric tumor samples and 31 normal gastric tissue samples, were selected from the GEO database. The platforms of these two datasets were identical to GPL570 (Affymetrix Human Genome U133 Plus 2.0 Array). We simultaneously obtained clinical information and gene expression profiles from patients by collecting GC information and non-cancerous samples from the TCGA database (TCGA-STAD) and GSE84437 (433 GC patients). The flowchart for the bioinformatics analysis is shown in Fig. 1.

### Data preprocessing and DEG identification

The “affy” package of R (version 3.6.3 http://r-project.org/), which allows exploratory analysis of oligonucleotide arrays, was used to read CEL files from the GEO database. Two professional bioinformatics analysts carried out the data preprocessing, including background correction, data normalization, removing batch effects, combining normal and tumor group data, ID transform gene symbols, and probe supplemental missing values [11]. Then, we identified DEGs using the “limma” package from Bioconductor [12]. Only genes with |log^FC(fold-change^)| > 2 and adj. *P* < 0.01 were selected. The volcano plot and Venn diagram were generated using “ggplot2” and “Venn diagram” packages, respectively.

### Identification of prognosis genes

Based on all the annotated genes (56,536 genes), the prognosis-related genes were identified using the “survival” package. The prognosis model was established using the Cox model of “Risk scores = ∑*coef* ∗ *Exp*(*genes*) ” in the “survival” package and optimized using the AIC value. The patients with TCGA with risk scores above the median were defined as the “high-risk group”, and the remaining patients were defined as the “low-risk group”. Singular and multiple factor analysis were utilized to estimate the independence and validity of the prognosis model.

### Clinical relevance and GSEA enrichment analysis

The correlations between the final filtered genes and clinical parameters were explored using TCGA, which included stage, T stage, age, grade, M stage, and N stage. Subsequently, the samples were divided into high and low expression groups, and GSEA was conducted to link genes with likely pathways [13]. Gene set permutations were performed 1000 times for each analysis. Based on the premise of FDR < 0.25 and NOM *P*-value < 0.05, we selected the enriched pathways of interest.

### Immune infiltration analysis

CIBERSORT, an analytical tool developed by Newman et al., uses gene expression data to estimate the abundance of member cell types in a mixed cell population [14]. We used the “CIBERSORT” package in R software to analyze possible associations between the genes and immune cells. Then TIMER, a comprehensive tool that systematically analyzes the infiltration of immune cells in various cancers, was used to analyze the relationships among the identified genes and five immune evaluation points (TOX, CD274, PDCD1LG2, CTLA4, and PDCD1) [15]. Additionally, we analyzed the association between ALDH3A2 copy number alterations and the STAD infiltration level. Finally, we used the cBioportal database to analyze the correlation between copy number alterations and the gene mRNA levels [16].

### Immunohistochemical staining

IHC staining was carried out on tissue sections obtained from 140 paraffin-embedded GC samples. Ten micron-thick sections of GC tissue were mounted on glass microscope slides, deparaffinized in xylene, and then rehydrated in a graded alcohol series. Antigen retrieval was performed at a high temperature using a water bath. The sections were cooled, rinsed, and endogenous peroxidases were quenched using 3% H2O2. After incubation in 5% BSA for 45 min at room temperature, the sections were incubated overnight in the ALDH3A2 antibody (dilution: 1:350; Abcam, city, state) at 4 °C. The sections were washed and incubated in secondary antibody for 60 min at room temperature. The antibody staining was visualized using the Dako EnVision System (Dako, Glostrup, Denmark). The IHC staining results were analyzed and scored by two pathologists who were blinded to the sources of the clinical samples. A semi-quantitative integration method was used to analyze the area and intensity of staining [17]. The proportion of cells that stained positive for ALDH3A2 was scored as 1 = 0 ~ 10%, 2 = 10% ~ 25%, 3 = 50% ~ 75%, and 4 = 75% ~ 100%. The intensity of staining was scored as 0 = no staining, 1 = weak staining, 2 = moderate staining, and 3 = strong staining. The final IHC score was calculated by multiplying one score by the other. Scores larger than six were regarded as a high score, and scores equal to or less than six were considered to be a low score.

### Quantitative PCR (qPCR)

Patient tissues used for PCR analysis were obtained from the Shanghai Pudong Hospital of Fudan University. This study was allowed by the Ethics Committee of the Shanghai Pudong Hospital of Fudan University. All patients had approved for the use of clinical tissues for research purposes. Total RNA was isolated using Trizol (Invitrogen). PrimeScript RT Master Mix (Takara, JPN) was used for first-strand cDNA synthesis. For the analysis of the ALDH3A2 mRNA levels, qPCR was performed using SYBR Green according to the manufacturer’s instructions (Applied Biosystems, USA). The primers that were used included: ALDH3A2, forward: 5-CTTGGAATTACCCCTTCGTTCTC-3; ALDH3A2, reverse: 5-TCCTGGTCTAAATACTGAGGGAG-3; PDCD1, forward: 5-ACGAGGGACAATAGGAGCCA-3; PDCD1, reverse: 5-GGCATACTCCGTCTGCTCAG-3; PDCD1LG2, forward: 5-ACCCTGGAATGCAACTTTGAC-3; PDCD1LG2, reverse: 5- AAGTGGCTCTTTCACGGTGTG-3; CTLA4, forward: 5-GCCCTGCACTCTCCTGTTTTT-3; CTLA4, reverse: 5-GGTTGCCGCACAGACTTCA-3; GAPDH, forward: 5-ACCACAGTCCATGCCATCAC-3; GAPDH, reverse: 5-TCCACCACCCTG TTGCTGTA-3.

### Protein extraction and Western blotting

Total proteins were extracted from human GC tissues using Western and IP lysis buffer (Beyotime, P0013; Beijing, China). The protein concentrations were measured using a BCA reagent kit (Pierce, 23,227). The proteins were resolved with 8–12% SDS-PAGE gels, then blotted onto polyvinylidene fluoride (PVDF) membranes. The membranes were blocked in TBS/0.1% Tween-20 (TBST) containing 5% powdered skim milk for 1 h at room temperature (RT). Primary antibodies, ALDH3A2, PDCD1, PDCD1LG2, CTLA4, and GAPDH (AtaGenix, Wuhan, China), were diluted to concentrations of 1:300 or 1:2000 before incubation with the membranes for 2 h at RT. Then the membranes were incubated in secondary antibodies [anti-rabbit or anti-mouse IgG (H + L) biotinylated antibodies (CST, USA)] for 2 h at RT.

### RNA interference studies

RNA interference of ALDH3A2 was carried out using small interfering RNA (siRNA). HGC-27 and MGC-803 cells were transfected with control siRNA and siRNA-ALDH3A2 using Lipofectamine 3000 (Invitrogen). The target sequence used for siRNA against ALDH3A2 was 5-GCATTGCACCCGACTATAT − 3. Western blots and qPCR were used to evaluate the efficiency of the siRNA interference.

### Statistical analysis

All statistical analyses and Kaplan-Meier survival curves were conducted using R software 3.6.3 [18]. *P* < 0.05 was deemed statistically significant. The relevance between ALDH3A2 and overall survival (OS) and other clinical variables were analyzed using multivariate Cox analysis. The area under the ROC curve (AUC value) was regarded as excellent for survival predictions when the value was greater than 0.7, and acceptable when the value was greater than 0.6. All the microscopy images were obtained in 300 dpi.

## Results

### Identification of DEGs in STAD

**Fig. 2:**
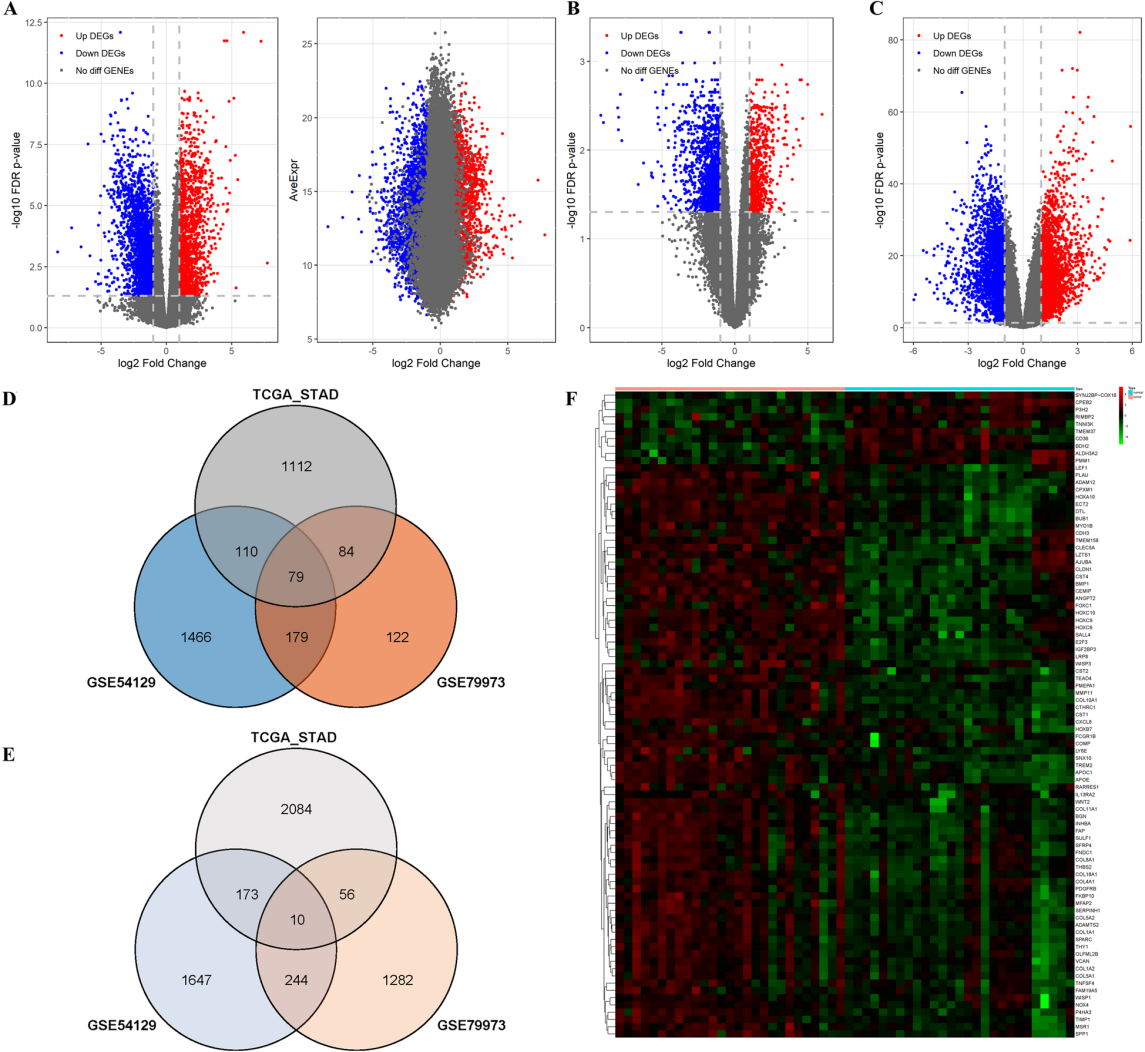
Identification of DEGs shared between the three databases. **a** The volcano plot of TCGA-STAD; **b** The volcano plot of GSE54129; **c** The volcano plot of GSE79973; **d** The venn diagram of up-regulated DEGs; **e** The venn diagram of down-regulated DEGs; **f** The heatmap of all 89 DEGs in TCGA-STAD. Abbreviations: TCGA, The Cancer Genome Atlas; GEO, Gene Expression Omnibus; DEGs, Differentially expressed genes

We compared the gene expression profiles between the cancerous and adjacent normal tissues using the GSE (GSE54129, GSE79973) and TCGA databases, with |log^FC(fold-change^)| > 2 and adj. *P* < 0.01. We identified 79 up-regulated and 10 down-regulated overlapping genes, which are shown as a volcano plot (Fig. 2a, b, c) and Venn diagram (Fig. 2d, e). These 89 differential genes also were mapped as a heatmap using the data from TCGA-STAD (Fig. 2f). The 79 upregulated and 10 downregulated DEGs are listed in Table 1.

**Table 1:** A total of 89 DEGs were identified from the TCGA and GEO datasets, with 79 up-regulated and 10 down-regulated

DEGs	Gene names
Down-regulated	SYNJ2BP-COX16, CD36, P3H2, TNNI3K, ALDH3A2, TMEM37, BDH2, PMM1, RIMBP2, CPEB2
Up-regulated	OLFML2B, SPARC, CLDN1, HOXA10, COMP, CDH3, PLAU, SALL4, CLEC5A, IL13RA2, FAM19A5, LZTS1, PDGFRB, SFRP4, FNDC1, COL4A1, ADAM12, COL8A1, AJUBA, HOXB7, LY6E, CEMIP, THY1, SERPINH1, APOE, ECT2, HOXC9, WNT2, HOXC6, MSR1, RARRES1, INHBA, COL1A1, COL1A2, BMP1, COL5A1, TNFSF4, P4HA3, COL5A2, NOX4, FKBP10, COL18A1, CXCL8, WISP3, SNX10, TREM2, HOXC10, WISP1, ADAMTS2, PMEPA1, COL10A1, CPXM1, TIMP1, TEAD4, BGN, MMP11, VCAN, DTL, FOXC1, COL11A1, LEF1, THBS2, LRP8, CST4, CST2, CST1, SPP1, E2F3, IGF2BP3, FCGR1B, BUB1, CTHRC1, ANGPT2, SULF1, MYO1B, TMEM158, FAP, APOC1, MFAP2

### Identification of the prognosis genes

**Fig. 3:**
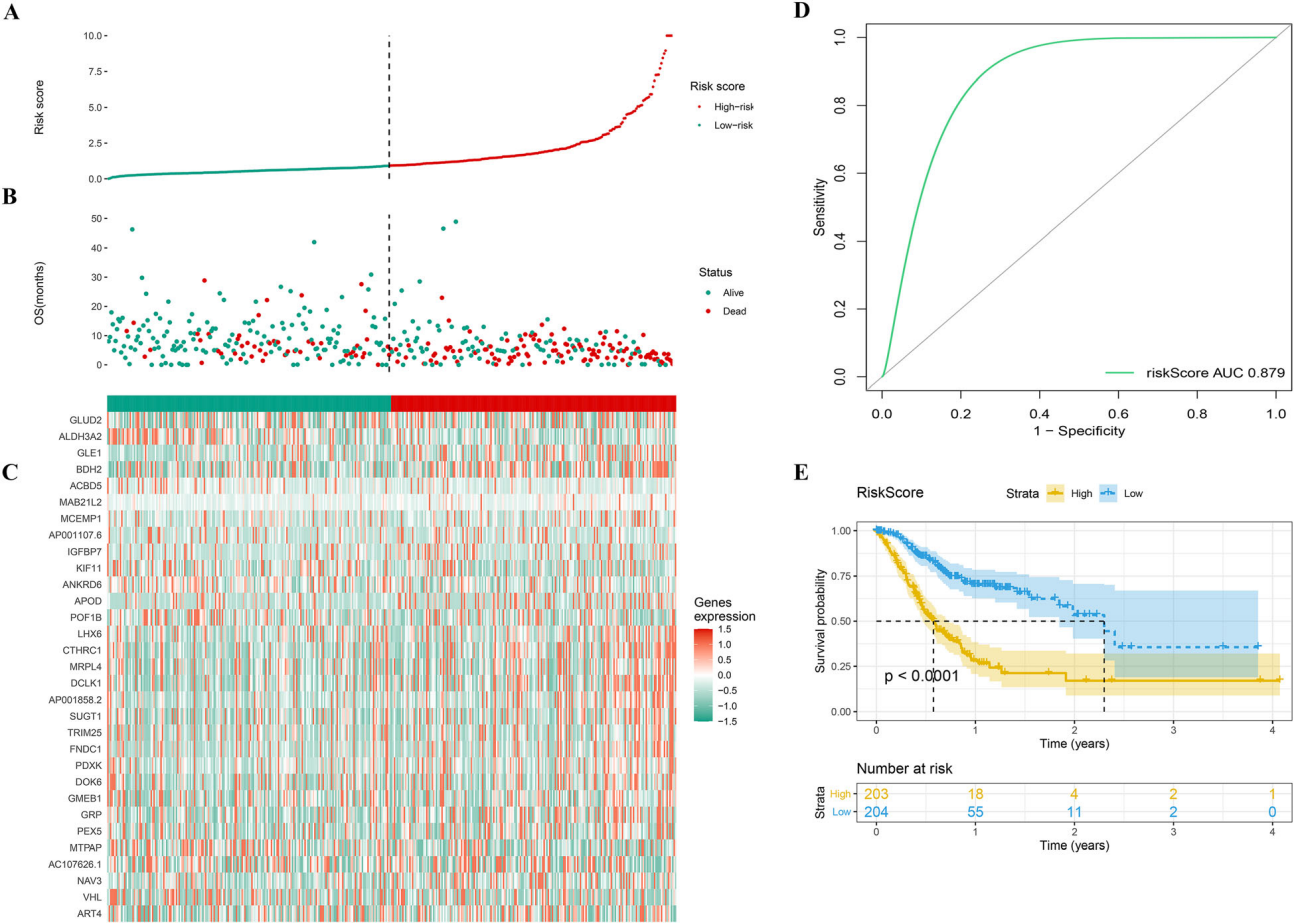
Visualization of prognosis-model in TCGA patients. **a** The high and low risk group of TCGA patients; **b** The survival status of high and low risk group; **c** The heatmap of prognosis-model genes in high and low risk group; **d** The ROC curve of prognosis-model; **e** The Kaplan-Meier survival curve of prognosis model. Abbreviations: TCGA, The Cancer Genome Atlas

We combined the gene expression matrix with the survival data from the TCGA patients and identified 1672 prognosis-related genes using the FM test and single factor Cox analysis (Table S1). After optimization using the “AIC” value, 31 genes were chosen to establish a prognostic model (Table S2). Each patient was placed in a high- or low-risk group based on risk scores computed using the “ ∑*coef* ∗ *Exp*(*genes*) ” formula (Fig. 3a, b). Figure 3c shows the expression of these 31 genes in the high- and low-risk groups. The area under the ROC curve (AUC value) was 0.879 (> 0.7), which proved the effectiveness of our prognosis model (Fig. 3d). Patients with high-risk scores exhibited a significantly worse prognosis compared to patients with low-risk scores (*p* < 0.0001) (Fig. 3e).

### Identification of candidate genes

**Fig. 4:**
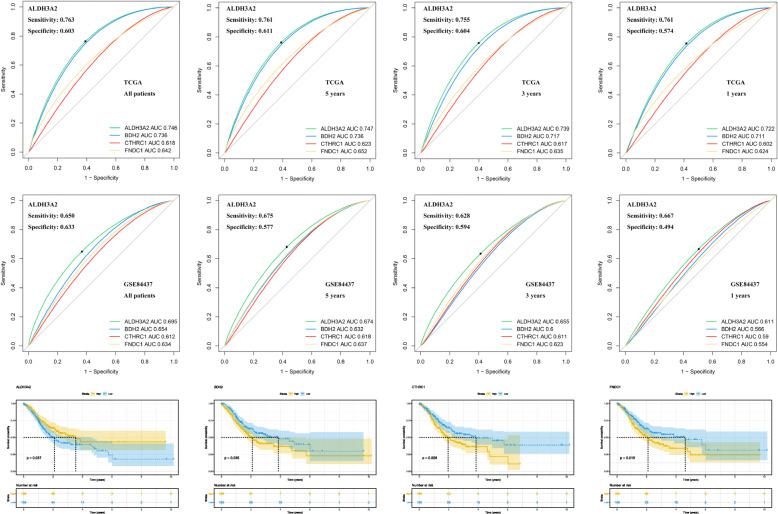
Sensitivity, specificity and predictive value in survival of ALDH3A2, BDH2, CTHRC1 and FNDC1. **a** Sensitivity, specificity and predictive value of these four genes in TCGA; **b** Sensitivity, specificity and predictive value of these four genes in GSE84437; **c** The Kaplan-Meier survival curve of these four genes in TCGA. Abbreviations: TCGA, The Cancer Genome Atlas

Comparison of the 89 differential genes and 31 prognostic genes identified four genes (ALDH3A2, BDH2, CTHRC1, and FNDC1) that were likely to have an important role in the development and progression of STAD. Figure 4a-b illustrates the sensitivity, specificity, and the AUC value for these four genes concerning their ability to predict the prognosis. Among these genes, ALDH3A2 exhibited the best results, and was selected for further analysis (TCGA, All patients: AUC = 0.746; 5 years: AUC = 0.747; 3 years: AUC = 0.739; 1 year: AUC = 0.722; GSE84437, All patients: AUC = 0.695; 5 years: AUC = 0.674; 3 years: AUC = 0.655; 1 years: AUC = 0.611). The KM survival curves for OS for these four genes are shown in Fig. 4c. Of these genes, the expression of ALDH3A2 was highly associated with a better prognosis. However, BDH2, CTHRC1, and FNDC1 indicated a worse prognosis.

### Clinical correlation and GSEA analysis

**Fig. 5:**
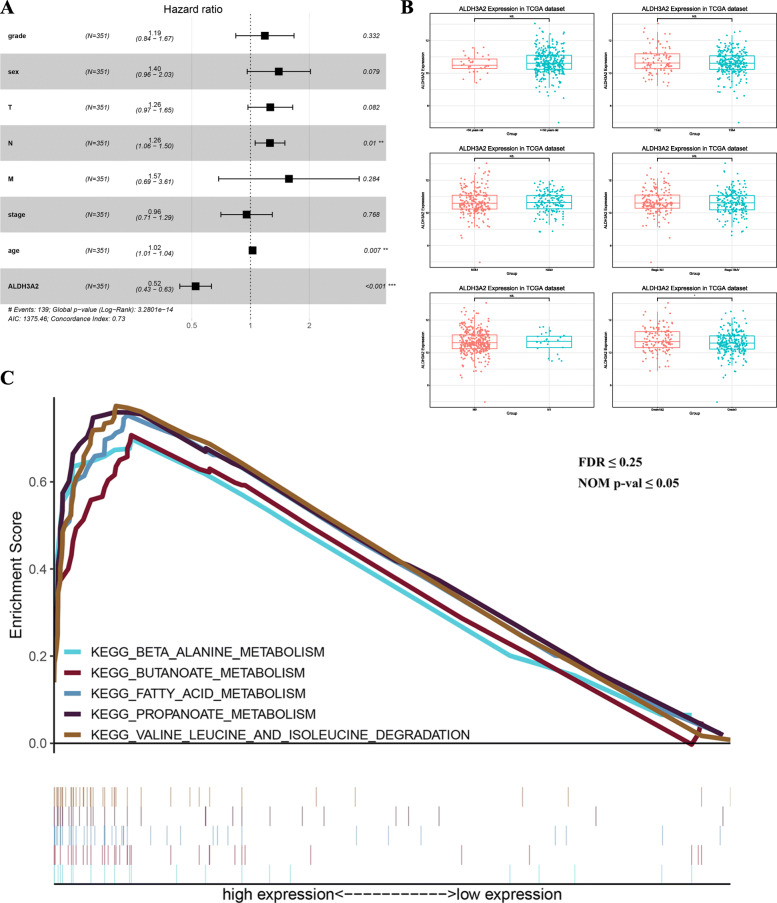
Clinical correlation and GSEA enrichment analysis of ALDH3A2. **a** Multivariate analysis of ALDH3A2 and clinical parameters; **b** The relevance between clinical characteristics and ALDH3A2 in TCGA patients; **c** S GSEA enrichment analysis of ALDH3A2. Abbreviations: GSEA, Gene Set Enrichment Analysis; TCGA, The Cancer Genome Atlas

The results of the multivariate Cox analysis revealed that age, N stage, and ALDH3A2 independently correlated with OS. This observation indicated that ALDH3A2 was an independent prognosis factor of STAD (Fig. 5a). To learn more about the impact of ADLH3A2 gene expression on GC, we examined the association of its expression with clinical characteristics in TCGA patients (Fig. 5b). However, the association was not significant except for the tumor grades (*P* < 0.05). To further explore the biological functions of ALDH3A2 in GC, we performed a GSEA enrichment analysis on the high and low ALDH3A2 expression datasets. As seen in Fig. 5c, the ALDH3A2 high expression phenotype, the signaling pathways of β-alanine metabolism, butanoate metabolism, fatty acid metabolism, propanoate metabolism, and valine leucine and isoleucine degradation were enriched (FDR < 0.25 and NOM *P*-value < 0.05).

### ALDH3A2 acts as an immune-related gene in STAD

**Fig. 6:**
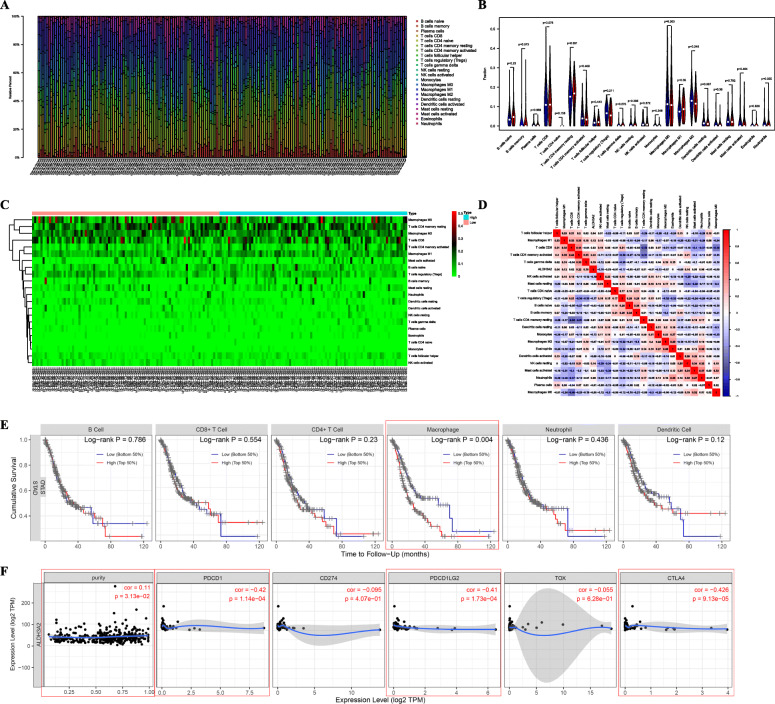
Tumor immune correlation analysis of ALDH3A2. **a** Relationship between ALDH3A2 expression and immune cells in TCGA patients; **b**&**c** Comparison between ALDH3A2 high and low expression in immune cells; **d** The co-expression relationship of diverse immune cells; **e** The association between immune cells and prognosis in TIMER website; **f** The association between ALDH3A2 and immune checkpoints. Abbreviations: TCGA, The Cancer Genome Atlas

The “CIBERSORT” package in R software and TIMER website was used to investigate the relationship between ALDH3A2 and tumor immunity (Fig. 6). One hundred seventy-eight samples met the criteria for immune infiltration analysis (Fig. 6a). These samples were divided into high and low ALDH3A2 groups (Fig. 6b, c). The results revealed that, compared with the low expression group, M1-type macrophages were highly expressed in the ALDH3A2 high expression group (Fig. 6b). The co-expression heatmap of diversified immune cells seen in Fig. 6d shows that CD4 memory resting T cells might be negatively associated with CD8 T cells, and neutrophils might be positively correlated with activated mast cells in STAD. We also examined the relationship between immune cell expression and survival, and discovered that elevated numbers of macrophages might predict a worse prognosis in STAD (*P* = 0.004; Fig. 6e). The immunological checkpoint analysis indicated that TOX, CD274, PDCD1LG2, CTLA4, and PDCD1 play a pivotal role in immunotherapy. Therefore, we analyzed the association between ALDH3A2 and these checkpoint-related genes (Fig. 6f). Interestingly, we found that ALDH3A2 co-expression might be negatively correlated with the PDCD1, PDCD1LG2, and CTLA4 genes, and positively associated with tumor purity. Furthermore, as seen in Figures S1 and S2, we found that ALDH3A2 copy number alterations might have an appreciable impact on the level of immune cell infiltration and mRNA expression. These results suggest that ALDH3A2 might influence the immune cell infiltration level through alterations in copy number, affecting the prognosis of STAD. In conclusion, ALDH3A2 showed a potential value for STAD remission and immunotherapy.

### High ALDH3A2 expression in GC tissues is associated with better survival

**Fig. 7:**
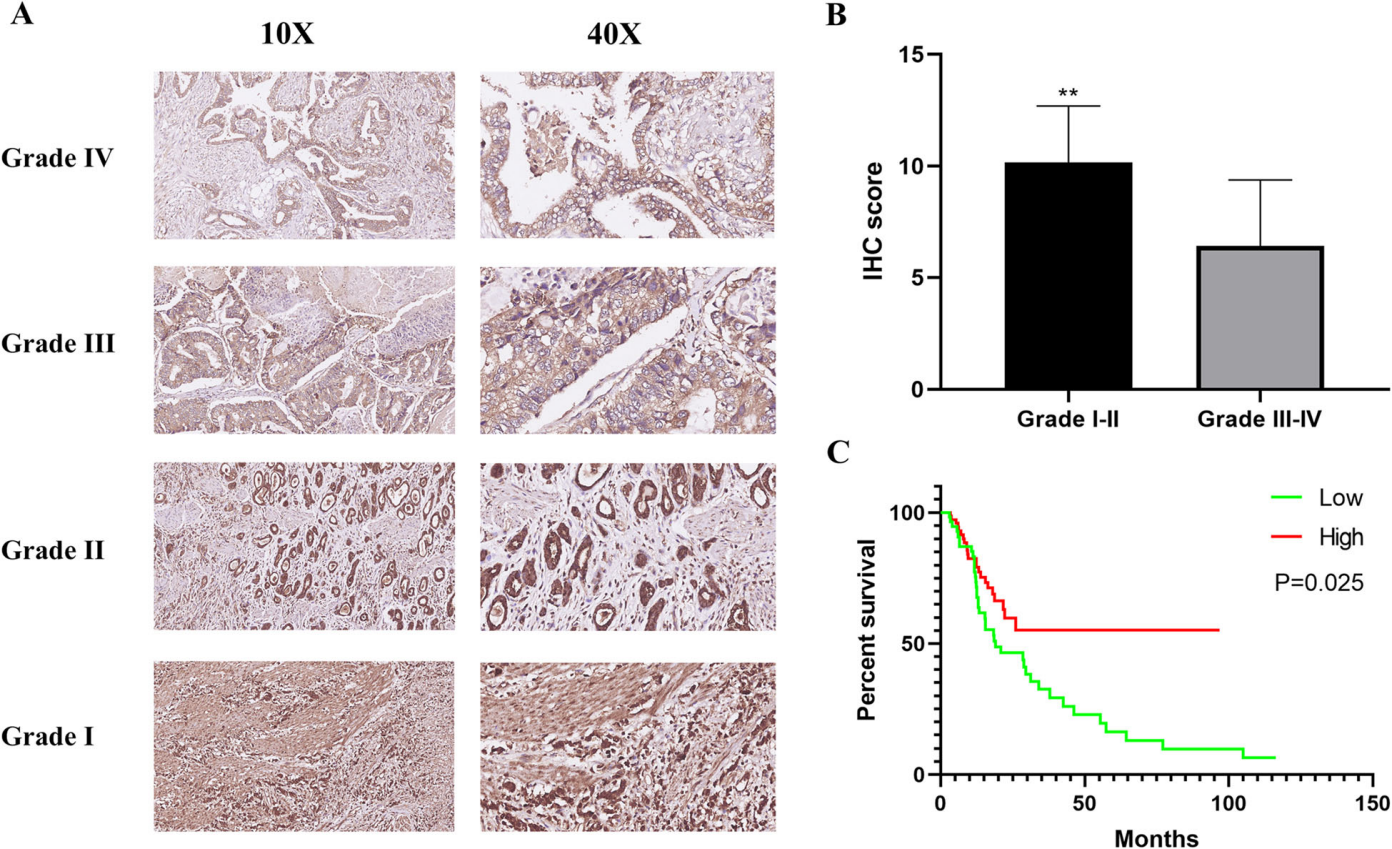
High ALDH3A2 expression in gastric cancer tissue correlates with good patients survival. **a** Representative images of ALDH3A2 expression in different grades of gastric cancer; **b** IHC score in different grades of gastric cancer; **c** Kaplan-Meier overall survival curves for all 140 patients with gastric cancer stratified with low and high expression of ALDH3A2

IHC was used to reveal ALDH3A2 expression in 140 paraffin-embedded human GC samples. Of the 140 GC samples, 8 cases were identified as grade I, 48 cases as grade II, 68 cases as grade III, and 16 cases as grade IV (Table 2). Compared with the high-grade GC cases (grades III and IV), ALDH3A2 was over-expressed in low-grade GC cases (grades I and II) (Fig. 7a-b). Kaplan-Meier survival curves demonstrated that the OS of patients with low expression of ALDH3A2 was significantly shorter than patients with high ALDH3A2 expression (Fig. 7c, *P* < 0.05).

**Table 2:** Clinicopathological characteristics of patient samples and expression of ALDH3A2 in gastric cancer

Characteristics of ALDH3A2	Number of cases (%)	ALDH3A2		***P***.value
		Low^**a**^	High^**b**^	
**Age (y)**				
> = 60	79	21	58	0.072
< 60	81	39	42	
Gender				
Male	106	48	58	0.302
Female	34	12	22	
**T classification**				
T1	12	7	5	0.502
T2	26	10	16	
T3	103	43	60	
**N classification**				
N0	62	26	36	0.456
N1	40	20	20	
N2	12	6	6	
N3	26	8	18	
**Grade**				
Grade I	8	2	6	< 0.001
Grade II	48	11	32	
Grade III	68	43	25	
Grade IV	16	12	4	
**Clinical stage**				
Stage I	30	12	18	0.724
Stage II	72	34	38	
Stage III	38	14	24	

^a^ scores <=6; ^b^ scores> 6

### The expression of ALDH3A2 was negatively correlated with PDCD1, PDCD1LG2, and CTLA-4

**Fig. 8:**
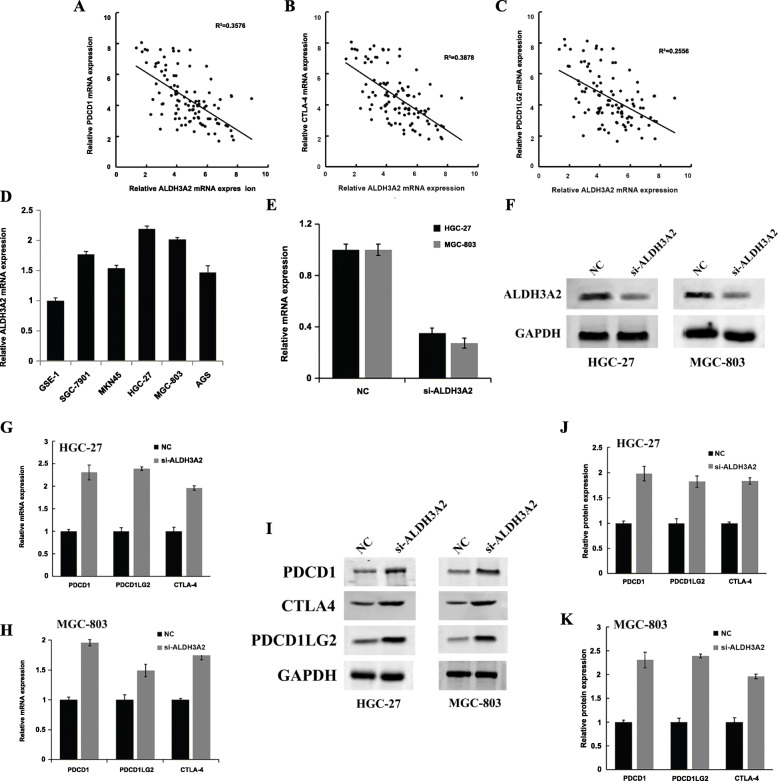
The expresasion of ALDH3A2 was negatively correlated with PDCD1, PDCD1LG2, and CTLA-4. **a** The negative correlation between ALDH3A2 and PDCD1 by qPCR; **b** The negative correlation between ALDH3A2 and CTLA-4 by qPCR; **c** The negative correlation between ALDH3A2 and PDCD1LG2 by qPCR; **d** qPCR of ALDH3A2 mRNA expression in GES-1, SGC-7901, MKN45, HGC-27, MGC-803 and AGS cell lines; **e** qPCR of indicated cells transfected with ALDH3A2-RNAi-vector, ALDH3A2-RNAi; **f** Western blotting of indicated cells transfected with ALDH3A2-RNAi-vector, ALDH3A2-RNAi; **g** qPCR revealed that downregulation of endogenous ALDH3A2 significantly decreased the mRNA level of PDCD1, CTLA-4 and PDCD1LG2 in HGC-27 cell lines; **h** qPCR revealed that downregulation of endogenous ALDH3A2 significantly decreased the mRNA level of PDCD1, CTLA-4 and PDCD1LG2 in MGC-803 cell lines; **i**-**k** Western blotting revealed that downregulation of endogenous ALDH3A2 significantly decreased the protein level of PDCD1, CTLA-4 and PDCD1LG2 in indicated cell lines

We used qPCR to analyze the ALDH3A2, PDCD1, PDCD1LG2, and CTLA-4 mRNA expression levels in 52 tumor tissues, which indicated a negative spatial correlation between ALDH3A2 and PDCD1 (Fig. 8a, *R*^2^ = 0.3576), PDCD1LG2 (Fig. 8b, *R*^2^ = 0.3878), and CTLA-4 (Fig. 8c, *R*^2^ = 0.2556). HGC-27 and MGC-803 cell lines exhibited the highest expression of ALDH3A2 and were selected for siRNA interference (Fig. 8d). Western blotting and qPCR revealed the successful knockdown of ALDH3A2 with siRNA interference (Fig. 8e-f). The Western blots revealed that, in the HGC-27 and MGC-803 cell lines, the knockdown of ALDH3A2 significantly increased the mRNA expression of PDCD1, PDCD1LG2, and CTLA-4 (Fig. 8g-h), which also was verified at the protein level (Fig. 8i-k). Full-length blots/gels are presented in Figure S3.

## Discussion

As the third most common cause of cancer-associated deaths, GC is a severe health problem worldwide. Despite the increase in early screening and planned prevention, the GC patients frequently are identified only towards the end of their terminal illness. These observations underscore the urgency of identifying new biomarkers for the diagnosis and prognosis of GC [19].

We gained a deeper understanding of GC gene expression through the present study by analyzing multiple gastric cancer data sets (GSE54129, GSE79973, and TCGA-STAD). A total of 89 DEGs were identified with 79 that were up-regulated, and 10 that were down-regulated. Moreover, 31 prognosis genes were identified using multivariate Cox analysis, followed by AIC optimization. Four genes, ALDH3A2, BDH2, CTHRC1, and FNDC1, were identified through analysis of the intersection of the 89 DEFs and 31 prognosis genes. We identified genes that could be used to predict the development of GC, guide therapy strategies, and might be novel prognostic biomarkers.

We explored the predictive value of ALDH3A2, BDH2, CTHRC1, and FNDC1 in prognosis (sensitivity, specificity, and AUC). ALDH3A2 was selected for further analysis. ALDH3A2, which is in the aldehyde dehydrogenase 3 family, member A2, is critically important in the detoxification of aldehydes generated by alcohol metabolism and lipid peroxidation, and mutations in this gene cause Sjogren-Larsson syndrome [20]. Few studies have focused on the role of ALDH3A2 in GC. Thus, to further study the effect of ALDH3A2 in GC, we examined the clinical features and conducted GSEA analysis in the high and low ALDH3A2 groups. ALDH3A2 had little effect on the clinical characteristics; only the grade of the tumor showed a significant correlation. Based on the GSEA analysis, several metabolic pathways were enriched, indicating that high expression of ALDH3A2 might improve the prognosis of GC by regulating metabolism. Even though metabolomics has great potential to help elucidate the complex mechanisms involved in the pathogenesis of disease, it remains relatively underutilized in studies of GC. By analyzing the aqueous metabolite liquid using chromatography-mass spectrometry, Tsai et al. found distinctive metabolomic profiles for GC compared to adjacent normal tissue [21]. Also, using chromatography-mass spectrometry, Liang et al. found that urine metabolic profiles were useful in detecting GC, which also might help to understand the underlying mechanisms of the pathogenesis of GC [22]. Our results elucidated the significance of ALDGH3A2 in GC metabolomics.

Considering the connections between metabolism and immunity, we evaluated the effect of ALDH3A2 on immune cell infiltration. Our results revealed that ALDH3A2 remarkably increased the numbers of M1 macrophages in the tumors. M1 macrophages (or activated macrophages), release pro-inflammatory cytokines, and induce an anti-tumor immune response that kills tumor cells and inhibits the formation of tumor lymphatics [23]. Although the specific mechanism is unclear, the positive regulatory effect of ALDH3A2 on M1 macrophages may partly explain the more positive prognosis of GC patients with high ALDH3A2 expression.

Finally, significant negative co-expression correlations were found among immune checkpoints, including PDCD1 (or PD-1), PDCD1LG2 (or PD-L2), CTLA4, and ALDH3A2. As a new field in tumor treatment, therapy that targets immune checkpoints has provided tremendous breakthroughs in cancer therapeutics [24]. Concerning GC, the influence of immune checkpoints on the prognosis is broad and complicated [25]. A previous study conducted by Kono et al. argued that the increased frequency of PD-1 positive macrophages might lead to a worse prognosis for GC patients [26]. Meanwhile, another cohort study from Egg et al. reported an elevated cancer risk in patients with CTLA-4 dysfunction [27]. We believe that ALDH3A2 may affect the development of GC as well as patient survival by affecting immune checkpoints such as PDCD1, PDCD1LG2, and CTLA4.

Furthermore, despite the overall robust statistical evidence generated by this analysis, this study lacked in vivo and in vitro investigations of the related mechanisms. Consequently, based on the direction provided by our results, our future studies will focus on a more in-depth analysis of the function and mechanisms of ALDH3A2, using a series of cellular, tissue, and animal experiments.

## Conclusions

In summary, from serial bioinformatics analysis of the TCGA, GSE database and IHC staining, we found that ALDH3A2 could effectively predict the prognosis of GC patients and might become an independent prognostic biomarker. Also, the interaction between ALDH3A2 and metabolism, M1 macrophages, and immune checkpoints (PDCD1, PDCD1LG2, and CTLA4) might underlie the prognostic impact.

## Supplementary information

**Additional file 1: Supplementary Figure S1.** The relation between ALDH3A2 copy number variation and infiltration level. Abbreviations: *, *P* < 0.05; **; *P* < 0.01; ***; *P* < 0.001.

**Additional file 2: Supplementary Figure S2.** The relationship between the copy number of ALDH3A2 and its mRNA level.

**Additional file 3: Supplementary Figure S3.** Full-length blots/gels of Fig. 8f&i are presented in Supplementary Figure S3. The cropped blots were marked with red frame (Photoshop cc 2018).

**Additional file 4: Table S1.** The 1672 prognosis-related genes.

**Additional file 5: Table S2.** The 31 genes of prognosis model.

## Data Availability

All data generated is included in the present article. Additional supporting material is contained in *Additional files*. The dataset has been uploaded to Figshare: https://figshare.com/articles/dataset/Original_file/13031699; DOI 10.6084/m9.figshare.13031699.v1. The open-acess data is available through the following URL – GSE54129 (https://www.ncbi.nlm.nih.gov/geo/query/acc.cgi?acc=GSE54129); GSE79973 (https://www.ncbi.nlm.nih.gov/geo/query/acc.cgi?acc=GSE79973); GSE84437 (https://www.ncbi.nlm.nih.gov/geo/query/acc.cgi?acc=GSE84437); GPL570 (https://www.ncbi.nlm.nih.gov/geo/query/acc.cgi?acc=GPL570); TCGA-STAD (https://portal.gdc.cancer.gov/).

## Abbreviations

GC: Gastric carcinoma
DEGs: Differentially expressed genes
GEO: Gene Expression Omnibus
TCGA: The Cancer Genome Atlas
FALDH: Fatty aldehyde dehydrogenase
GSEA: Gene set enrichment analysis
STAD: Stomach adenocarcinomas
VEGF: Vascular endothelial growth factor
MMP: Matrix metalloproteinase
siRNAs: Small interfering RNAs
miRNAs: MicroRNAs
LncRNAs: Long non-coding RNAs
IHC: Immunohistochemical
RT: Room temperature
