# Correction to: Identification of ALDH3A2 as a novel prognostic biomarker in gastric adenocarcinoma using integrated bioinformatics analysis

**DOI:** 10.1186/s12885-020-07659-7

**Published:** 2020-11-24

**Authors:** Zhenhua Yin, Dejun Wu, Jianping Shi, Xiyi Wei, Nuyun Jin, Xiaolan Lu, Xiaohan Ren

**Affiliations:** 1 Department of Digestive, Shanghai Pudong Hospital, Fudan University Pudong Medical Center, 2800 Gongwei Road, Shanghai, 201399 China; 2 Department of General Surgery, Shanghai Pudong Hospital, Fudan University Pudong Medical Center, 2800 Gongwei Road, Shanghai, 201399 China; 3 The State Key Lab of Reproductive, Department of Urology, The First Affiliated Hospital of Nanjing Medical University, Nanjing, 210029 China

**Correction to: BMC Cancer 20, 1062 (2020)**

**https://doi.org/10.1186/s12885-020-07493-x**

Following publication of the original article [1], the authors reported an error in the funding statement. The funding number was mistakenly given as PWZxk 2017–17 instead of PWZxk 2017–27.

The original article [1] has been corrected.
