# Supplementary figures and images for: Identification of ALDH3A2 as a novel prognostic biomarker in gastric adenocarcinoma using integrated bioinformatics analysis
Source: BMC Cancer. 2020 Nov 4;20:1062. doi: 10.1186/s12885-020-07493-x (PMC7640415; doi:10.1186/s12885-020-07493-x)

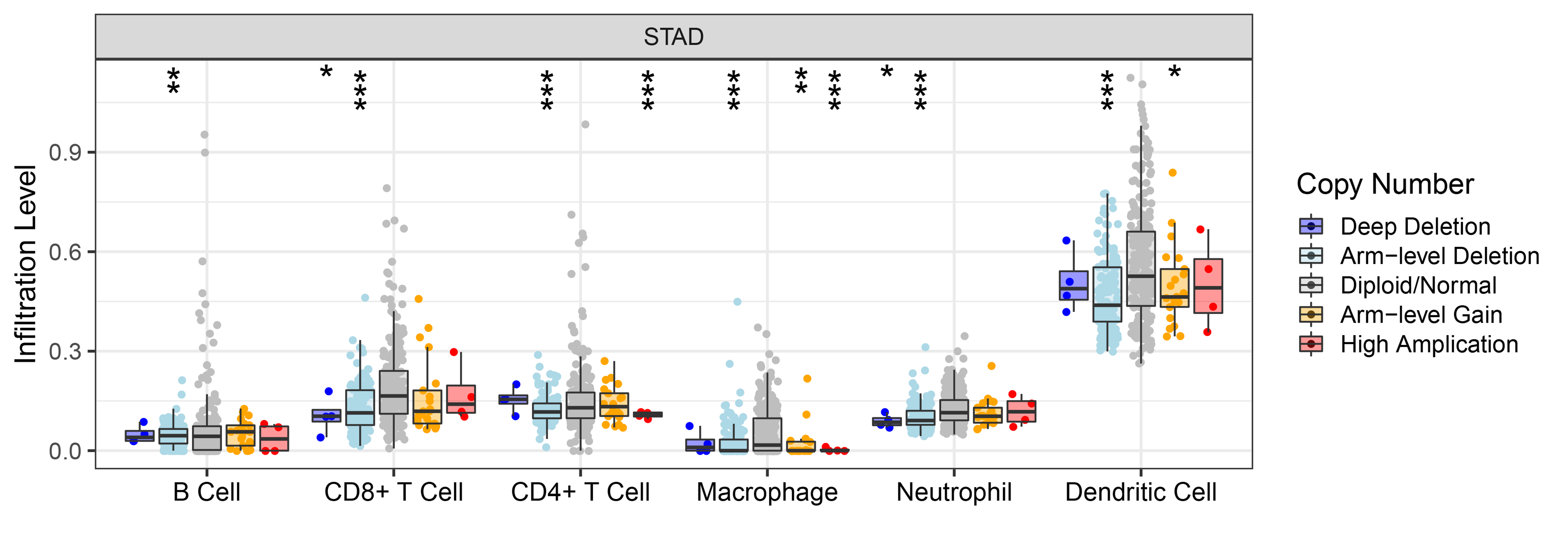

Supplement: Supplementary file 1 — Additional file 1: Supplementary Figure S1. The relation between ALDH3A2 copy number variation and infiltration level. Abbreviations: *, P < 0.05; **; P < 0.01; ***; P < 0.001. [file 12885_2020_7493_MOESM1_ESM.tif]

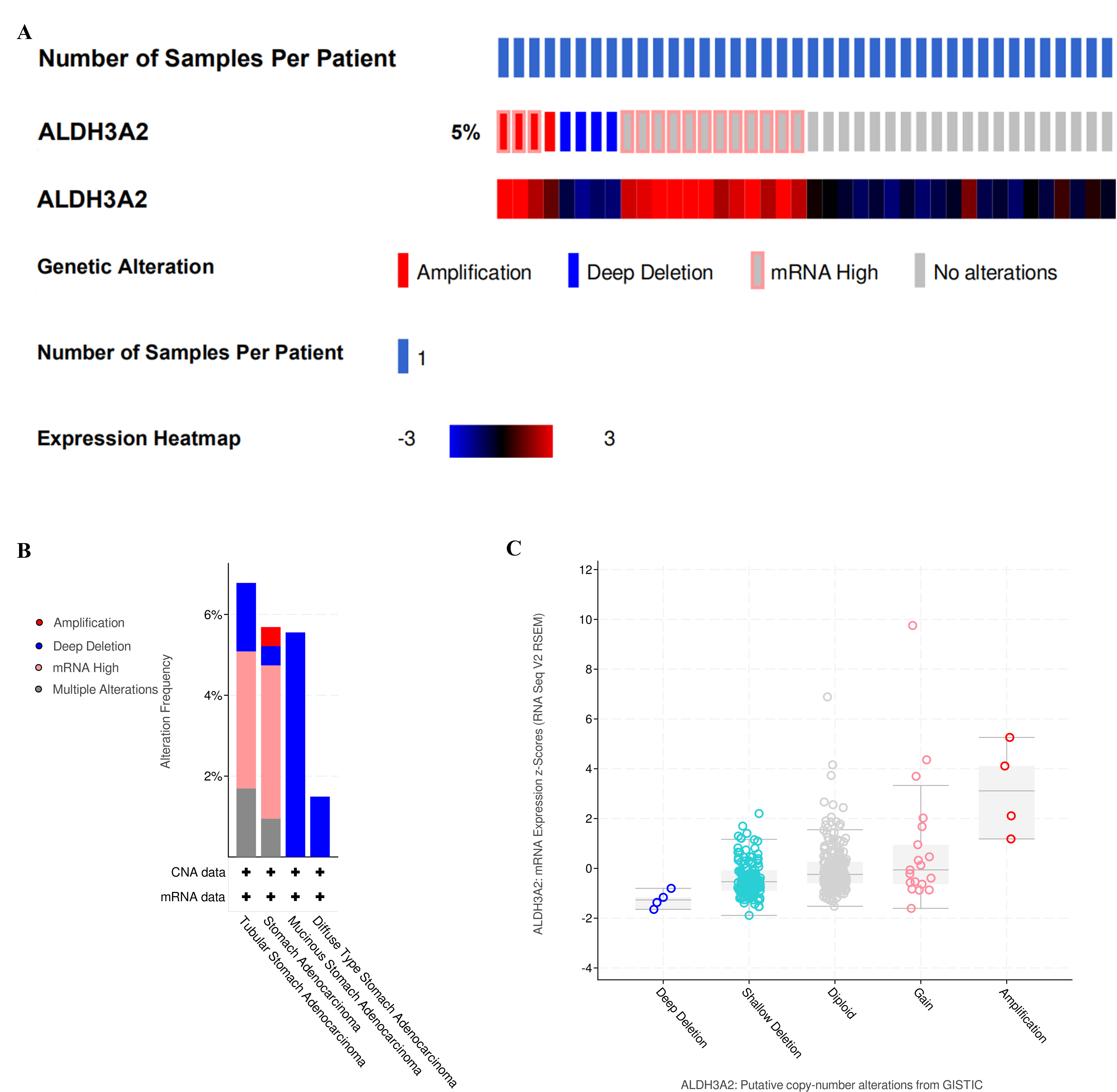

Supplement: Supplementary file 2 — Additional file 2: Supplementary Figure S2. The relationship between the copy number of ALDH3A2 and its mRNA level. [file 12885_2020_7493_MOESM2_ESM.tif]

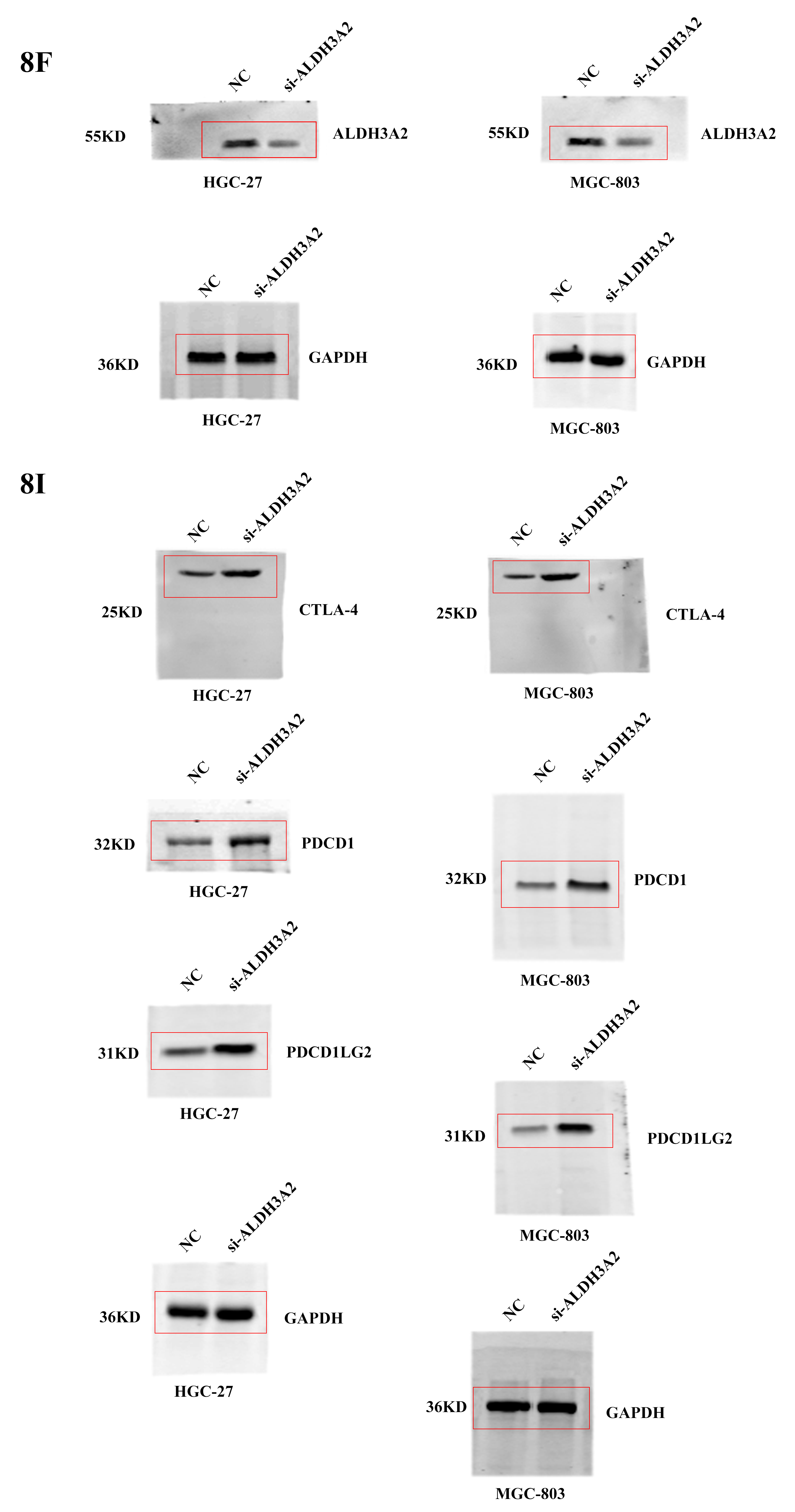

Supplement: Supplementary file 3 — Additional file 3: Supplementary Figure S3. Full-length blots/gels of Fig. 8f&i are presented in Supplementary Figure S3. The cropped blots were marked with red frame (Photoshop cc 2018). [file 12885_2020_7493_MOESM3_ESM.tif]
