# Supplementary material for: Identification of ALDH3A2 as a novel prognostic biomarker in gastric adenocarcinoma using integrated bioinformatics analysis
Source: BMC Cancer. 2020 Nov 4;20:1062. doi: 10.1186/s12885-020-07493-x (PMC7640415; doi:10.1186/s12885-020-07493-x)
Supplement: Supplementary file 4 — Additional file 4: Table S1. The 1672 prognosis-related genes. [file 12885_2020_7493_MOESM4_ESM.docx]

| **Table S1. The 1672 prognosis-related genes** | | | | |
| --- | --- | --- | --- | --- |
| Gene | HR | HR.95L | HR.95H | pvalue |
| C1orf210 | 0.97 | 0.95 | 0.99 | 0.01 |
| PDGFRB | 1.01 | 1.00 | 1.01 | 0.02 |
| OR5AR1 | 13131.92 | 22.51 | 7662238.33 | 0.00 |
| BDH2 | 1.11 | 1.03 | 1.20 | 0.01 |
| AC119501.1 | 114.70 | 2.90 | 4530.30 | 0.01 |
| KRTAP2-2 | 41.87 | 23.96 | 72.80 | 0.00 |
| NGLY1 | 0.82 | 0.69 | 0.97 | 0.02 |
| PVT1 | 0.90 | 0.82 | 0.99 | 0.02 |
| AC092958.2 | 6.19 | 1.29 | 29.73 | 0.02 |
| MARCKS | 1.02 | 1.01 | 1.03 | 0.00 |
| IGFBP7 | 1.00 | 1.00 | 1.00 | 0.01 |
| AC244098.2 | 1067.30 | 2.05 | 554872.74 | 0.03 |
| ACTG1P13 | 117.05 | 2.76 | 4968.82 | 0.01 |
| RPS15AP34 | 10.66 | 2.41 | 47.10 | 0.00 |
| AC084759.1 | 280.04 | 9.15 | 8575.20 | 0.00 |
| BSDC1 | 0.95 | 0.90 | 0.99 | 0.02 |
| SSPN | 1.04 | 1.01 | 1.08 | 0.02 |
| BDH2P1 | 2.89 | 1.06 | 7.88 | 0.04 |
| RECK | 1.18 | 1.07 | 1.30 | 0.00 |
| H3P36 | 1.31 | 1.03 | 1.67 | 0.03 |
| RAD54L | 0.89 | 0.81 | 0.97 | 0.01 |
| MIR7853 | 8.13 | 1.56 | 42.40 | 0.01 |
| DAZAP1 | 0.94 | 0.90 | 0.99 | 0.01 |
| AL954722.1 | 1.91 | 1.05 | 3.48 | 0.03 |
| AC098799.3 | 8.52 | 1.64 | 44.19 | 0.01 |
| PSMC1P3 | 8.68 | 1.29 | 58.36 | 0.03 |
| GLE1 | 0.95 | 0.92 | 0.98 | 0.00 |
| AL606516.1 | 5155.54 | 19.39 | 1371086.74 | 0.00 |
| AC107067.1 | 6.89 | 1.72 | 27.52 | 0.01 |
| RN7SKP31 | 2812.00 | 9.08 | 870804.92 | 0.01 |
| LINC01960 | 13.86 | 1.11 | 173.07 | 0.04 |
| GRP | 1.07 | 1.02 | 1.13 | 0.01 |
| AP000894.2 | 3.90 | 1.58 | 9.66 | 0.00 |
| MRGPRX11P | 518.19 | 7.79 | 34487.68 | 0.00 |
| DCLK1 | 1.09 | 1.04 | 1.14 | 0.00 |
| AP001858.2 | 1.10 | 1.03 | 1.18 | 0.01 |
| PLXDC2 | 1.04 | 1.01 | 1.07 | 0.00 |
| PGBD4P4 | 34.66 | 2.84 | 422.80 | 0.01 |
| NAV2-AS4 | 290.56 | 4.99 | 16923.86 | 0.01 |
| AC010170.1 | 8.40 | 1.71 | 41.23 | 0.01 |
| AL589684.1 | 24.21 | 3.85 | 152.34 | 0.00 |
| TMEM161A | 0.94 | 0.90 | 0.99 | 0.01 |
| AC107024.1 | 13.54 | 2.48 | 73.83 | 0.00 |
| ANKRD6 | 1.39 | 1.07 | 1.81 | 0.02 |
| ST6GALNAC3 | 1.33 | 1.09 | 1.61 | 0.00 |
| HIGD1AP5 | 6.69 | 1.58 | 28.39 | 0.01 |
| AP001107.6 | 0.34 | 0.16 | 0.71 | 0.00 |
| NIPA2P5 | 5.81 | 1.61 | 20.91 | 0.01 |
| PDXK | 0.97 | 0.94 | 1.00 | 0.04 |
| AL591504.1 | 36.18 | 3.08 | 424.43 | 0.00 |
| EVX2 | 3.33 | 1.08 | 10.34 | 0.04 |
| CHCHD2P7 | 53.45 | 3.69 | 774.38 | 0.00 |
| NACAP6 | 1115.55 | 32.39 | 38418.12 | 0.00 |
| GMEB1 | 0.77 | 0.62 | 0.96 | 0.02 |
| CTSLP6 | 106.82 | 2.25 | 5068.98 | 0.02 |
| RBMS1P1 | 1.36 | 1.07 | 1.74 | 0.01 |
| SLC8B1 | 0.93 | 0.87 | 1.00 | 0.04 |
| DAB2IP | 0.98 | 0.96 | 1.00 | 0.02 |
| ADGRF5 | 1.04 | 1.00 | 1.09 | 0.04 |
| PPIAP49 | 2.19 | 1.25 | 3.83 | 0.01 |
| TRIM26 | 0.95 | 0.92 | 0.98 | 0.00 |
| LINC02161 | 25.00 | 3.50 | 178.73 | 0.00 |
| HMG20B | 0.96 | 0.93 | 0.99 | 0.02 |
| AL132996.1 | 12.20 | 1.15 | 129.51 | 0.04 |
| APOD | 1.00 | 1.00 | 1.00 | 0.00 |
| MBOAT4 | 1.17 | 1.04 | 1.31 | 0.01 |
| AC092376.2 | 9.52 | 2.67 | 33.96 | 0.00 |
| OR2T12 | 2353.67 | 14.91 | 371629.47 | 0.00 |
| Z99289.2 | 15.81 | 3.17 | 78.80 | 0.00 |
| RNA5SP94 | 6.12 | 1.91 | 19.65 | 0.00 |
| CHAC1 | 0.96 | 0.92 | 1.00 | 0.03 |
| TRIM25 | 0.94 | 0.90 | 0.98 | 0.00 |
| AC023141.5 | 578.99 | 1.33 | 251982.48 | 0.04 |
| OR52M1 | 261.87 | 9.51 | 7209.22 | 0.00 |
| GPR42 | 1332.14 | 7.23 | 245458.22 | 0.01 |
| ANTXR1 | 1.01 | 1.00 | 1.02 | 0.02 |
| GULP1 | 1.08 | 1.02 | 1.13 | 0.00 |
| AC107613.1 | 3874.77 | 13.06 | 1149225.30 | 0.00 |
| AC079248.3 | 14.72 | 1.92 | 112.71 | 0.01 |
| SNORA30B | 2.23 | 1.14 | 4.34 | 0.02 |
| RPL12P34 | 27.50 | 1.70 | 445.80 | 0.02 |
| TMEM164 | 0.97 | 0.94 | 0.99 | 0.01 |
| CRAMP1 | 0.81 | 0.65 | 1.00 | 0.05 |
| CD36 | 1.05 | 1.01 | 1.09 | 0.01 |
| AC007351.3 | 6.05 | 1.43 | 25.65 | 0.01 |
| TCEA2 | 1.06 | 1.00 | 1.12 | 0.05 |
| RASAL1 | 0.95 | 0.91 | 0.99 | 0.02 |
| AL136295.3 | 0.01 | 0.00 | 0.62 | 0.03 |
| OR4A9P | 185.64 | 4.78 | 7213.44 | 0.01 |
| AC090049.1 | 42.90 | 2.37 | 775.45 | 0.01 |
| KIF11 | 0.97 | 0.94 | 1.00 | 0.03 |
| OSBPL1A | 1.09 | 1.03 | 1.16 | 0.00 |
| AXL | 1.02 | 1.01 | 1.03 | 0.00 |
| AC010872.1 | 10.46 | 2.36 | 46.39 | 0.00 |
| AL356490.1 | 5.59 | 1.25 | 25.01 | 0.02 |
| EHMT1 | 0.87 | 0.77 | 0.99 | 0.03 |
| LINC02791 | 74.07 | 5.77 | 951.28 | 0.00 |
| MRPL4 | 0.97 | 0.95 | 0.99 | 0.01 |
| AC023194.1 | 11.56 | 3.53 | 37.85 | 0.00 |
| LINC02684 | 3.43 | 1.12 | 10.46 | 0.03 |
| SAR1AP4 | 1467.42 | 50.04 | 43028.88 | 0.00 |
| C3orf62 | 0.71 | 0.55 | 0.92 | 0.01 |
| ARHGEF39 | 0.89 | 0.81 | 0.99 | 0.03 |
| LHX6 | 1.41 | 1.14 | 1.76 | 0.00 |
| AC060788.1 | 10.32 | 1.25 | 85.14 | 0.03 |
| F7 | 1.09 | 1.02 | 1.17 | 0.01 |
| ZBTB10 | 1.09 | 1.01 | 1.17 | 0.03 |
| AL645608.3 | 1.28 | 1.01 | 1.62 | 0.04 |
| AL117355.1 | 112.25 | 2.89 | 4361.68 | 0.01 |
| ARMCX1 | 1.07 | 1.02 | 1.12 | 0.00 |
| AP001528.2 | 1.29 | 1.03 | 1.62 | 0.03 |
| AL356387.2 | 22.30 | 1.94 | 256.01 | 0.01 |
| ATP1B2 | 1.10 | 1.01 | 1.20 | 0.04 |
| MTCYBP24 | 13.24 | 1.28 | 137.39 | 0.03 |
| AL354811.1 | 1.58 | 1.00 | 2.49 | 0.05 |
| ST3GAL6 | 1.35 | 1.12 | 1.62 | 0.00 |
| AC015908.5 | 14.19 | 2.17 | 92.83 | 0.01 |
| AL157359.3 | 33.57 | 2.44 | 462.25 | 0.01 |
| SUGT1 | 0.88 | 0.78 | 0.99 | 0.03 |
| AP001124.1 | 22.15 | 1.55 | 317.47 | 0.02 |
| LINC01423 | 11.62 | 1.58 | 85.32 | 0.02 |
| AC107626.1 | 6.73 | 1.68 | 27.02 | 0.01 |
| AC117944.1 | 10.16 | 1.55 | 66.54 | 0.02 |
| VCAN | 1.02 | 1.01 | 1.03 | 0.00 |
| OR5H3P | 55.57 | 2.66 | 1161.84 | 0.01 |
| C8orf82 | 0.98 | 0.95 | 1.00 | 0.05 |
| BORA | 0.90 | 0.81 | 1.00 | 0.04 |
| SH3BGRL | 1.00 | 1.00 | 1.01 | 0.02 |
| MCEMP1 | 1.18 | 1.07 | 1.30 | 0.00 |
| RN7SL813P | 15.31 | 2.44 | 95.90 | 0.00 |
| AP001033.4 | 1.22 | 1.01 | 1.48 | 0.04 |
| AC002486.2 | 2.34 | 1.15 | 4.78 | 0.02 |
| SNORD114-23 | 2.44 | 1.08 | 5.52 | 0.03 |
| AP000705.2 | 11.97 | 1.10 | 130.39 | 0.04 |
| INCENP | 0.93 | 0.90 | 0.97 | 0.00 |
| ART4 | 1.29 | 1.03 | 1.63 | 0.03 |
| OLFML2B | 1.01 | 1.00 | 1.02 | 0.00 |
| ARHGEF17 | 1.03 | 1.00 | 1.07 | 0.05 |
| AC100775.1 | 886.51 | 22.53 | 34883.29 | 0.00 |
| AP003122.6 | 112.86 | 2.95 | 4316.03 | 0.01 |
| KRTAP20-1 | 10.75 | 1.76 | 65.57 | 0.01 |
| GPR26 | 10.27 | 1.10 | 95.63 | 0.04 |
| TRIM51DP | 14.34 | 2.05 | 100.43 | 0.01 |
| ITGAV | 1.02 | 1.01 | 1.04 | 0.00 |
| DOK6 | 2.16 | 1.26 | 3.69 | 0.01 |
| S100A12 | 1.01 | 1.00 | 1.01 | 0.02 |
| FP671120.3 | 0.95 | 0.91 | 1.00 | 0.04 |
| OR4A7P | 58.94 | 3.48 | 997.95 | 0.00 |
| PABPC1P11 | 1419.46 | 16.59 | 121448.73 | 0.00 |
| RFX5 | 0.96 | 0.93 | 0.99 | 0.01 |
| IGHVIII-11-1 | 21.95 | 5.17 | 93.23 | 0.00 |
| UBE2Q2P11 | 4.42 | 1.25 | 15.71 | 0.02 |
| LRP12 | 1.24 | 1.01 | 1.52 | 0.04 |
| OR5AL1 | 216.76 | 2.54 | 18493.07 | 0.02 |
| RNA5SP517 | 5.13 | 1.85 | 14.26 | 0.00 |
| ELOCP23 | 89.23 | 6.02 | 1321.60 | 0.00 |
| TMEM200A | 1.06 | 1.02 | 1.10 | 0.00 |
| WNT10B | 1.13 | 1.01 | 1.25 | 0.03 |
| ZRSR2P1 | 0.06 | 0.00 | 0.67 | 0.02 |
| AL603840.1 | 40.29 | 3.33 | 488.22 | 0.00 |
| DYNLL1P3 | 2.35 | 1.18 | 4.71 | 0.02 |
| OR5M5P | 51.13 | 3.04 | 859.43 | 0.01 |
| AC011195.1 | 0.66 | 0.46 | 0.96 | 0.03 |
| DDR2 | 1.03 | 1.01 | 1.05 | 0.01 |
| RNU6-367P | 14.33 | 2.30 | 89.27 | 0.00 |
| OR5BL1P | 66.33 | 3.74 | 1175.92 | 0.00 |
| AC073136.2 | 3.54 | 1.06 | 11.83 | 0.04 |
| PLCE1P1 | 9.31 | 1.15 | 75.64 | 0.04 |
| ATP8B2 | 1.05 | 1.01 | 1.09 | 0.01 |
| POF1B | 0.99 | 0.98 | 1.00 | 0.02 |
| SRPX2 | 1.06 | 1.02 | 1.10 | 0.00 |
| AC098483.3 | 11.39 | 1.38 | 94.14 | 0.02 |
| AP005202.2 | 19.97 | 1.03 | 385.86 | 0.05 |
| AC078865.1 | 10.71 | 1.17 | 97.69 | 0.04 |
| RNA5SP272 | 4.67 | 1.49 | 14.60 | 0.01 |
| RN7SL728P | 4.51 | 1.14 | 17.91 | 0.03 |
| Z93241.1 | 0.31 | 0.10 | 0.96 | 0.04 |
| KC877982.1 | 1.84 | 1.35 | 2.49 | 0.00 |
| AC007688.1 | 0.85 | 0.74 | 0.98 | 0.03 |
| CPNE8 | 1.30 | 1.16 | 1.46 | 0.00 |
| LINC00602 | 18.48 | 2.39 | 142.86 | 0.01 |
| RNU6-117P | 1.29 | 1.12 | 1.49 | 0.00 |
| AC105914.2 | 95.17 | 5.65 | 1603.83 | 0.00 |
| AC097535.1 | 36.86 | 2.02 | 672.70 | 0.01 |
| PTP4A2P2 | 2.37 | 1.23 | 4.56 | 0.01 |
| SMARCA4 | 0.97 | 0.94 | 1.00 | 0.02 |
| NT5E | 1.02 | 1.01 | 1.03 | 0.00 |
| SNCG | 1.03 | 1.01 | 1.04 | 0.00 |
| AC114744.1 | 2.81 | 1.45 | 5.44 | 0.00 |
| OMD | 1.04 | 1.01 | 1.07 | 0.01 |
| BRI3BP | 0.95 | 0.92 | 0.98 | 0.00 |
| AL031779.1 | 4.56 | 1.25 | 16.66 | 0.02 |
| AP002833.1 | 13.09 | 1.36 | 126.18 | 0.03 |
| CCDC178 | 6.65 | 2.05 | 21.55 | 0.00 |
| ADGRE5 | 0.98 | 0.96 | 1.00 | 0.02 |
| GLUD2 | 1.29 | 1.08 | 1.54 | 0.01 |
| AC007041.1 | 3.12 | 1.01 | 9.60 | 0.05 |
| LMNB2 | 0.98 | 0.96 | 0.99 | 0.00 |
| METTL15P3 | 34.32 | 2.19 | 538.24 | 0.01 |
| PEX5 | 0.90 | 0.84 | 0.97 | 0.00 |
| OLFM3 | 18.81 | 1.52 | 233.20 | 0.02 |
| ZC2HC1A | 1.11 | 1.01 | 1.22 | 0.02 |
| INPP5J | 0.82 | 0.69 | 0.96 | 0.02 |
| OR4A5 | 33.60 | 2.12 | 531.92 | 0.01 |
| SNAPC4 | 0.92 | 0.84 | 1.00 | 0.04 |
| PLPPR4 | 1.13 | 1.04 | 1.23 | 0.00 |
| FBN2 | 1.23 | 1.00 | 1.51 | 0.05 |
| LINC01705 | 1.09 | 1.01 | 1.19 | 0.03 |
| MAB21L2 | 1.10 | 1.02 | 1.20 | 0.02 |
| GOLGA6L5P | 5.82 | 1.29 | 26.36 | 0.02 |
| AC004585.2 | 13.64 | 1.47 | 126.89 | 0.02 |
| RNA5SP487 | 2.08 | 1.23 | 3.52 | 0.01 |
| PRXL2B | 0.99 | 0.98 | 1.00 | 0.02 |
| SNORD115-18 | 5.49 | 1.78 | 16.94 | 0.00 |
| OACYLP | 2.42 | 1.01 | 5.80 | 0.05 |
| MYL4 | 1.95 | 1.16 | 3.25 | 0.01 |
| SYN1 | 1.05 | 1.00 | 1.11 | 0.04 |
| MTPAP | 0.85 | 0.74 | 0.98 | 0.02 |
| DSCR10 | 2.51 | 1.55 | 4.06 | 0.00 |
| AP001342.1 | 171.92 | 12.37 | 2390.17 | 0.00 |
| OR7E23P | 29.38 | 1.88 | 458.18 | 0.02 |
| ITPRIP | 1.04 | 1.01 | 1.07 | 0.00 |
| SMIM10L2B | 1.38 | 1.00 | 1.89 | 0.05 |
| CASC22 | 5.03 | 1.09 | 23.16 | 0.04 |
| MTND4P17 | 242.52 | 6.35 | 9257.44 | 0.00 |
| GABRG1 | 80.82 | 7.13 | 916.06 | 0.00 |
| MIR514A2 | 74.58 | 1.15 | 4828.06 | 0.04 |
| AC010300.1 | 0.40 | 0.18 | 0.88 | 0.02 |
| VHL | 0.96 | 0.92 | 0.99 | 0.02 |
| AC138853.1 | 1080.53 | 5.10 | 228785.63 | 0.01 |
| MGAT5 | 0.97 | 0.95 | 1.00 | 0.03 |
| AC124319.1 | 0.82 | 0.70 | 0.97 | 0.02 |
| LINC02465 | 7.36 | 1.30 | 41.56 | 0.02 |
| STARD3NL | 1.08 | 1.00 | 1.17 | 0.05 |
| NAV3 | 1.52 | 1.06 | 2.17 | 0.02 |
| CYP4F34P | 1.15 | 1.00 | 1.31 | 0.05 |
| NCLP2 | 1968.91 | 5.38 | 720329.24 | 0.01 |
| AP001528.1 | 1.42 | 1.11 | 1.83 | 0.01 |
| RNA5SP262 | 4.63 | 1.06 | 20.24 | 0.04 |
| PRR15L | 0.99 | 0.99 | 1.00 | 0.00 |
| OR5AN2P | 7.89 | 1.43 | 43.50 | 0.02 |
| TRAJ61 | 2.15 | 1.41 | 3.29 | 0.00 |
| ACBD5 | 0.97 | 0.94 | 0.99 | 0.01 |
| AC007677.1 | 5.84 | 1.21 | 28.15 | 0.03 |
| DCDC2C | 14.77 | 2.72 | 80.11 | 0.00 |
| RNA5SP419 | 8.03 | 1.09 | 59.18 | 0.04 |
| PRICKLE1 | 1.39 | 1.18 | 1.63 | 0.00 |
| CKAP4 | 1.01 | 1.00 | 1.02 | 0.02 |
| AC010608.1 | 2.11 | 1.07 | 4.18 | 0.03 |
| RNU6-623P | 0.60 | 0.36 | 0.98 | 0.04 |
| AP005436.2 | 2378873.58 | 830.66 | 6812729262.00 | 0.00 |
| MIR4635 | 0.91 | 0.83 | 1.00 | 0.04 |
| TAF5 | 0.81 | 0.67 | 0.98 | 0.03 |
| PLCB3 | 0.98 | 0.97 | 1.00 | 0.01 |
| AL137067.1 | 9.18 | 2.52 | 33.43 | 0.00 |
| CLDN9 | 1.03 | 1.00 | 1.06 | 0.04 |
| ADGRG5 | 0.84 | 0.74 | 0.96 | 0.01 |
| CNTN4 | 1.37 | 1.10 | 1.69 | 0.00 |
| AC008056.2 | 2.65 | 1.03 | 6.83 | 0.04 |
| CALU | 1.01 | 1.00 | 1.02 | 0.01 |
| MIR5695 | 0.70 | 0.52 | 0.96 | 0.03 |
| AC091965.3 | 4.72 | 1.36 | 16.33 | 0.01 |
| LRFN5 | 2.93 | 1.07 | 8.02 | 0.04 |
| AC105105.2 | 21.36 | 5.45 | 83.66 | 0.00 |
| AC027369.2 | 72.61 | 3.92 | 1346.24 | 0.00 |
| PAXBP1P1 | 3182.78 | 44.30 | 228675.22 | 0.00 |
| AL022722.2 | 5.19 | 1.20 | 22.49 | 0.03 |
| SGPP2 | 0.99 | 0.98 | 1.00 | 0.05 |
| ATP13A1 | 0.97 | 0.93 | 1.00 | 0.05 |
| AL513533.1 | 10.59 | 1.23 | 91.51 | 0.03 |
| MIR3689F | 3.88 | 1.10 | 13.72 | 0.04 |
| AC138024.1 | 17.21 | 1.92 | 154.39 | 0.01 |
| SNORD114-7 | 1.79 | 1.16 | 2.77 | 0.01 |
| AC091770.1 | 11.23 | 2.00 | 63.06 | 0.01 |
| CTNNAL1 | 1.04 | 1.01 | 1.07 | 0.00 |
| GPR173 | 1.32 | 1.08 | 1.62 | 0.01 |
| NOS2P1 | 503.74 | 2.77 | 91513.74 | 0.02 |
| AL355574.1 | 0.81 | 0.70 | 0.94 | 0.01 |
| NTF6A | 381.48 | 13.94 | 10442.64 | 0.00 |
| NMNAT1 | 0.69 | 0.56 | 0.86 | 0.00 |
| OR11Q1P | 78.51 | 4.66 | 1322.62 | 0.00 |
| AP4B1 | 0.85 | 0.74 | 0.98 | 0.02 |
| STC1 | 1.01 | 1.01 | 1.02 | 0.00 |
| RNF180 | 1.25 | 1.01 | 1.55 | 0.04 |
| AL596327.1 | 79.55 | 4.70 | 1345.13 | 0.00 |
| SPINK14 | 1.91 | 1.31 | 2.80 | 0.00 |
| LPAR2 | 0.95 | 0.91 | 1.00 | 0.03 |
| ATG4D | 0.95 | 0.91 | 0.99 | 0.02 |
| AC009554.1 | 0.46 | 0.22 | 0.93 | 0.03 |
| AP002530.1 | 2123.40 | 66.69 | 67611.97 | 0.00 |
| AC117383.2 | 2.44 | 1.01 | 5.88 | 0.05 |
| PCDH12 | 1.12 | 1.01 | 1.23 | 0.03 |
| RNU4-66P | 2.25 | 1.07 | 4.74 | 0.03 |
| KRT18P56 | 1.73 | 1.02 | 2.94 | 0.04 |
| RNA5SP410 | 11.62 | 4.14 | 32.60 | 0.00 |
| AC008991.1 | 19.34 | 1.90 | 196.55 | 0.01 |
| VSTM4 | 1.10 | 1.02 | 1.20 | 0.02 |
| CYCSP22 | 16.29 | 1.62 | 163.73 | 0.02 |
| SLC9A3R2 | 1.02 | 1.00 | 1.03 | 0.02 |
| EIF3EP1 | 1.59 | 1.15 | 2.19 | 0.00 |
| AC008555.6 | 36.04 | 2.22 | 586.28 | 0.01 |
| RNA5SP210 | 2.04 | 1.06 | 3.92 | 0.03 |
| KLHDC8A | 1.31 | 1.02 | 1.69 | 0.04 |
| TTTY12 | 13410.78 | 19.90 | 9037063.67 | 0.00 |
| CBX3P6 | 243.35 | 3.49 | 16987.90 | 0.01 |
| AC023512.1 | 1.61 | 1.13 | 2.30 | 0.01 |
| LINC00449 | 0.41 | 0.20 | 0.84 | 0.02 |
| RN7SL310P | 19.85 | 1.49 | 264.42 | 0.02 |
| P4HA3 | 1.16 | 1.06 | 1.28 | 0.00 |
| AGT | 1.00 | 1.00 | 1.00 | 0.03 |
| MANCR | 1.17 | 1.05 | 1.29 | 0.00 |
| MISP | 1.00 | 0.99 | 1.00 | 0.01 |
| FARSA | 0.98 | 0.96 | 1.00 | 0.02 |
| FRG2DP | 9.53 | 1.41 | 64.65 | 0.02 |
| OR5B10P | 7.97 | 1.52 | 41.87 | 0.01 |
| MTCO2P23 | 19.07 | 2.74 | 132.58 | 0.00 |
| RNU6-298P | 0.70 | 0.50 | 0.99 | 0.05 |
| IGHVII-33-1 | 14.89 | 1.38 | 160.68 | 0.03 |
| LINC02853 | 14.40 | 2.88 | 71.91 | 0.00 |
| MEOX2 | 1.08 | 1.03 | 1.14 | 0.00 |
| NOS2P2 | 4.52 | 1.42 | 14.39 | 0.01 |
| RNU6-139P | 5.93 | 1.51 | 23.28 | 0.01 |
| RNU6-967P | 49.94 | 2.39 | 1043.01 | 0.01 |
| PKP2 | 0.98 | 0.96 | 1.00 | 0.04 |
| AC010723.1 | 26192.84 | 13.16 | 52116089.89 | 0.01 |
| AC127521.1 | 0.71 | 0.55 | 0.93 | 0.01 |
| AL391336.2 | 3.13 | 1.23 | 7.93 | 0.02 |
| PLA2R1 | 1.15 | 1.02 | 1.29 | 0.02 |
| AKT3 | 1.06 | 1.01 | 1.10 | 0.01 |
| OR52A5 | 27.84 | 1.46 | 532.55 | 0.03 |
| SORCS2 | 1.19 | 1.06 | 1.33 | 0.00 |
| MIR1179 | 6.06 | 1.91 | 19.22 | 0.00 |
| TRIM51JP | 6691.64 | 22.64 | 1977500.25 | 0.00 |
| METTL2A | 0.85 | 0.75 | 0.97 | 0.01 |
| RAMP2 | 1.01 | 1.00 | 1.02 | 0.02 |
| TLR5 | 1.13 | 1.01 | 1.27 | 0.04 |
| AL365434.1 | 4.03 | 1.05 | 15.52 | 0.04 |
| FTO | 1.16 | 1.03 | 1.30 | 0.01 |
| AC129507.1 | 3.79 | 1.81 | 7.96 | 0.00 |
| AC107021.1 | 5.06 | 1.96 | 13.05 | 0.00 |
| PATJ | 0.91 | 0.85 | 0.97 | 0.00 |
| AL049792.1 | 69.20 | 3.32 | 1442.10 | 0.01 |
| AC084361.1 | 9.41 | 1.84 | 48.03 | 0.01 |
| CDC37 | 0.98 | 0.97 | 0.99 | 0.00 |
| PDE1B | 1.41 | 1.17 | 1.71 | 0.00 |
| OSBPL9P1 | 216.52 | 5.42 | 8653.45 | 0.00 |
| SDF2 | 1.05 | 1.01 | 1.10 | 0.02 |
| AC008534.2 | 68.69 | 3.73 | 1264.27 | 0.00 |
| AAMP | 0.98 | 0.96 | 1.00 | 0.01 |
| MIR548F3 | 6.49 | 2.31 | 18.22 | 0.00 |
| NCLN | 0.98 | 0.97 | 0.99 | 0.00 |
| EGF | 1.47 | 1.19 | 1.80 | 0.00 |
| GPR4 | 1.08 | 1.00 | 1.15 | 0.04 |
| SNORC | 0.93 | 0.88 | 0.99 | 0.03 |
| AP001825.3 | 9.56 | 2.13 | 42.82 | 0.00 |
| AL162497.1 | 14.97 | 1.26 | 178.45 | 0.03 |
| AC044839.2 | 11.50 | 1.41 | 93.57 | 0.02 |
| AC010991.1 | 129.13 | 14.57 | 1144.75 | 0.00 |
| MIR3179-3 | 3758.70 | 1.31 | 10780947.03 | 0.04 |
| C8orf87 | 2.54 | 1.24 | 5.19 | 0.01 |
| CEP78 | 0.76 | 0.61 | 0.93 | 0.01 |
| AL513325.1 | 5.00 | 1.20 | 20.78 | 0.03 |
| CCR3 | 1.82 | 1.11 | 2.98 | 0.02 |
| TPMTP3 | 38.62 | 2.40 | 622.74 | 0.01 |
| AC021146.10 | 4.55 | 1.14 | 18.12 | 0.03 |
| SLC52A3 | 0.95 | 0.92 | 0.98 | 0.00 |
| OPN1SW | 1.18 | 1.03 | 1.35 | 0.02 |
| ELL2P2 | 531.92 | 8.44 | 33519.31 | 0.00 |
| LINC00315 | 30.49 | 1.91 | 487.58 | 0.02 |
| MSC-AS1 | 1.42 | 1.07 | 1.89 | 0.02 |
| ECSCR | 1.08 | 1.01 | 1.16 | 0.03 |
| POLR1A | 0.89 | 0.82 | 0.96 | 0.00 |
| RNU4-73P | 16.65 | 3.10 | 89.54 | 0.00 |
| NOP14 | 0.97 | 0.94 | 1.00 | 0.05 |
| ZNF22 | 1.09 | 1.03 | 1.15 | 0.00 |
| IGHVII-44-2 | 25.55 | 1.55 | 420.87 | 0.02 |
| AC006001.4 | 1.05 | 1.00 | 1.09 | 0.03 |
| AC006499.4 | 2277.81 | 15.85 | 327377.87 | 0.00 |
| IGHD5OR15-5A | 2.10 | 1.22 | 3.63 | 0.01 |
| SEMA5B | 1.29 | 1.03 | 1.62 | 0.03 |
| OR10J4 | 61.52 | 1.52 | 2492.43 | 0.03 |
| CC2D1A | 0.95 | 0.92 | 0.99 | 0.01 |
| ZNF562 | 0.84 | 0.73 | 0.97 | 0.02 |
| IGLVVI-22-1 | 12.15 | 2.10 | 70.12 | 0.01 |
| AGGF1P8 | 29.24 | 1.07 | 802.00 | 0.05 |
| AC080098.1 | 5.45 | 1.50 | 19.86 | 0.01 |
| KPNA7 | 1.05 | 1.01 | 1.10 | 0.01 |
| OR2M5 | 9888.01 | 22.03 | 4438928.05 | 0.00 |
| AC091044.1 | 0.07 | 0.01 | 0.71 | 0.02 |
| VEGFC | 1.09 | 1.02 | 1.17 | 0.01 |
| AC117409.1 | 2.85 | 1.33 | 6.09 | 0.01 |
| ERRFI1 | 1.01 | 1.00 | 1.02 | 0.03 |
| PLOD2 | 1.05 | 1.02 | 1.08 | 0.00 |
| LINC01941 | 16.81 | 1.38 | 205.39 | 0.03 |
| AC093831.1 | 309.80 | 7.99 | 12014.01 | 0.00 |
| IGFBP7-AS1 | 2.17 | 1.10 | 4.26 | 0.02 |
| AP005900.1 | 3.34 | 1.07 | 10.44 | 0.04 |
| AC016751.1 | 14.99 | 1.75 | 128.16 | 0.01 |
| LARP6 | 1.10 | 1.01 | 1.20 | 0.03 |
| AL391152.1 | 2.20 | 1.32 | 3.65 | 0.00 |
| RN7SL714P | 14.58 | 3.31 | 64.19 | 0.00 |
| AL354984.1 | 7.39 | 2.01 | 27.20 | 0.00 |
| AP006193.1 | 13.08 | 1.57 | 108.89 | 0.02 |
| AC044798.1 | 15.59 | 2.49 | 97.67 | 0.00 |
| AC091435.2 | 4.86 | 1.85 | 12.75 | 0.00 |
| AL390254.1 | 11.94 | 2.61 | 54.72 | 0.00 |
| ARPP21-AS1 | 74.56 | 3.31 | 1679.44 | 0.01 |
| PXDN | 1.02 | 1.00 | 1.04 | 0.02 |
| C1QBPP1 | 38.34 | 3.70 | 396.67 | 0.00 |
| IFNA2 | 2712.12 | 10.84 | 678718.73 | 0.01 |
| CDR2 | 1.07 | 1.01 | 1.12 | 0.01 |
| FAM181B | 1.26 | 1.01 | 1.58 | 0.04 |
| RNU6-512P | 2.45 | 1.37 | 4.38 | 0.00 |
| AKR1B1P7 | 7.24 | 1.06 | 49.43 | 0.04 |
| AL353804.1 | 0.76 | 0.59 | 0.99 | 0.04 |
| PICK1 | 0.91 | 0.82 | 1.00 | 0.04 |
| MOCS1 | 1.08 | 1.00 | 1.17 | 0.04 |
| CRISPLD2 | 1.02 | 1.00 | 1.03 | 0.02 |
| RNU6-1023P | 1.95 | 1.15 | 3.33 | 0.01 |
| IGHVIII-22-2 | 1.24 | 1.00 | 1.53 | 0.05 |
| AL162431.2 | 0.29 | 0.09 | 0.93 | 0.04 |
| HNRNPA3P8 | 32.27 | 2.04 | 511.13 | 0.01 |
| OR2J3 | 7.02 | 1.29 | 38.30 | 0.02 |
| RAMP1 | 1.01 | 1.00 | 1.01 | 0.05 |
| OR2X1P | 9170.15 | 35.40 | 2375653.58 | 0.00 |
| RN7SL589P | 0.69 | 0.50 | 0.96 | 0.03 |
| LNCOG | 2.03 | 1.03 | 3.97 | 0.04 |
| FAAH2 | 0.92 | 0.85 | 1.00 | 0.04 |
| TCN2 | 1.03 | 1.00 | 1.05 | 0.05 |
| RARB | 1.13 | 1.00 | 1.28 | 0.05 |
| EXOC7P1 | 361.11 | 3.76 | 34642.08 | 0.01 |
| AC021146.8 | 119.16 | 2.11 | 6719.28 | 0.02 |
| AP001880.2 | 109.96 | 3.54 | 3411.42 | 0.01 |
| MIR5590 | 2.49 | 1.10 | 5.64 | 0.03 |
| SLC12A7 | 0.99 | 0.98 | 1.00 | 0.02 |
| TTF2 | 0.88 | 0.78 | 0.99 | 0.04 |
| VEZT | 1.07 | 1.00 | 1.14 | 0.04 |
| OR4N5 | 45.89 | 1.99 | 1057.07 | 0.02 |
| GJA1P1 | 3.92 | 1.06 | 14.48 | 0.04 |
| C19orf25 | 0.90 | 0.82 | 1.00 | 0.04 |
| OR13C9 | 19.03 | 1.17 | 309.23 | 0.04 |
| AL355306.2 | 153.54 | 3.51 | 6708.73 | 0.01 |
| OR4K15 | 203.91 | 5.55 | 7496.98 | 0.00 |
| AC123786.1 | 7.52 | 1.40 | 40.44 | 0.02 |
| MEI4 | 2.49 | 1.21 | 5.13 | 0.01 |
| OR51S1 | 21.43 | 2.18 | 210.83 | 0.01 |
| AL121748.1 | 5.36 | 2.21 | 13.04 | 0.00 |
| MED22 | 0.91 | 0.83 | 0.99 | 0.03 |
| ITIH3 | 1.59 | 1.08 | 2.34 | 0.02 |
| AC138982.1 | 28.65 | 5.37 | 152.81 | 0.00 |
| HCAR1 | 1.11 | 1.02 | 1.21 | 0.02 |
| ERCC6L | 0.81 | 0.68 | 0.97 | 0.02 |
| LINC01628 | 652.62 | 3.23 | 131941.22 | 0.02 |
| TRMT112P2 | 2.78 | 1.31 | 5.91 | 0.01 |
| ERICH3-AS1 | 56.13 | 2.07 | 1525.54 | 0.02 |
| FRG2FP | 133.02 | 6.83 | 2591.37 | 0.00 |
| MRGPRX2 | 53.63 | 2.97 | 969.70 | 0.01 |
| L3MBTL3 | 1.18 | 1.00 | 1.38 | 0.05 |
| PCAT19 | 1.27 | 1.02 | 1.57 | 0.03 |
| SLC25A1P4 | 11.36 | 1.95 | 66.21 | 0.01 |
| RN7SL341P | 912.51 | 10.43 | 79843.75 | 0.00 |
| AL009181.2 | 3.58 | 1.01 | 12.65 | 0.05 |
| AL442644.1 | 98.12 | 2.65 | 3638.32 | 0.01 |
| AEBP1 | 1.00 | 1.00 | 1.00 | 0.05 |
| TFPI | 1.04 | 1.02 | 1.05 | 0.00 |
| UBAC1 | 0.94 | 0.90 | 0.99 | 0.02 |
| OR4C4P | 8420.76 | 13.87 | 5112538.69 | 0.01 |
| OR2J2 | 21.59 | 2.10 | 222.37 | 0.01 |
| ADAT3 | 0.85 | 0.78 | 0.93 | 0.00 |
| JAM3 | 1.03 | 1.00 | 1.06 | 0.03 |
| AC010528.1 | 5.67 | 1.32 | 24.26 | 0.02 |
| RPL23AP29 | 6.91 | 2.11 | 22.61 | 0.00 |
| NLRP3P1 | 6.48 | 2.22 | 18.91 | 0.00 |
| IFNA4 | 30758.63 | 31.11 | 30414219.65 | 0.00 |
| FAM171B | 1.15 | 1.03 | 1.29 | 0.02 |
| AC022616.3 | 11.77 | 2.17 | 63.92 | 0.00 |
| AC079163.1 | 13.03 | 1.44 | 118.24 | 0.02 |
| C1QL2 | 1.18 | 1.02 | 1.37 | 0.03 |
| MIR485 | 35.20 | 1.30 | 956.41 | 0.03 |
| RNA5SP103 | 5.75 | 1.98 | 16.71 | 0.00 |
| MARK2P10 | 59.18 | 2.47 | 1417.38 | 0.01 |
| CGB5 | 1.01 | 1.00 | 1.01 | 0.03 |
| CALB2 | 1.02 | 1.00 | 1.03 | 0.01 |
| MYB | 0.96 | 0.93 | 0.99 | 0.01 |
| LINC00305 | 3.91 | 1.38 | 11.09 | 0.01 |
| AP000812.2 | 2.44 | 1.05 | 5.69 | 0.04 |
| TIRAP | 0.85 | 0.75 | 0.97 | 0.01 |
| RPL12P7 | 2.35 | 1.02 | 5.38 | 0.04 |
| SOCS3 | 1.01 | 1.00 | 1.01 | 0.03 |
| CYCSP42 | 35.37 | 1.04 | 1199.70 | 0.05 |
| BEND6 | 1.98 | 1.22 | 3.19 | 0.01 |
| RGS5 | 1.01 | 1.00 | 1.01 | 0.01 |
| CATSPER1 | 1.19 | 1.04 | 1.38 | 0.01 |
| AC019176.1 | 7.80 | 1.67 | 36.40 | 0.01 |
| LRRTM4-AS1 | 8.52 | 1.27 | 57.00 | 0.03 |
| EDNRA | 1.05 | 1.01 | 1.08 | 0.00 |
| AC145141.2 | 22.78 | 1.36 | 383.00 | 0.03 |
| RNU1-150P | 2.73 | 1.20 | 6.22 | 0.02 |
| GAMT | 1.02 | 1.01 | 1.04 | 0.00 |
| MIR4509-2 | 28.46 | 1.38 | 588.62 | 0.03 |
| DSTNP4 | 6.25 | 1.70 | 22.93 | 0.01 |
| AC015468.3 | 16.76 | 2.48 | 113.18 | 0.00 |
| OR7H2P | 139.02 | 4.18 | 4625.25 | 0.01 |
| ATP6V1E1P1 | 2.81 | 1.07 | 7.38 | 0.04 |
| TSC22D3 | 1.01 | 1.00 | 1.02 | 0.03 |
| LRRC32 | 1.02 | 1.00 | 1.03 | 0.01 |
| AC011853.1 | 5.70 | 1.39 | 23.44 | 0.02 |
| AC109811.2 | 1088.22 | 7.88 | 150220.28 | 0.01 |
| AL355001.1 | 0.47 | 0.27 | 0.82 | 0.01 |
| AARS1P1 | 93.63 | 2.91 | 3012.43 | 0.01 |
| AC027682.6 | 1.54 | 1.02 | 2.33 | 0.04 |
| LINC02310 | 4.87 | 1.38 | 17.15 | 0.01 |
| PSMD12 | 0.95 | 0.89 | 1.00 | 0.05 |
| VCAN-AS1 | 12.92 | 3.15 | 53.06 | 0.00 |
| ADAMTS10 | 1.14 | 1.00 | 1.29 | 0.05 |
| FIGNL2-DT | 1.45 | 1.01 | 2.09 | 0.05 |
| AC008687.6 | 1.50 | 1.21 | 1.85 | 0.00 |
| AC099786.2 | 1383.97 | 22.98 | 83364.79 | 0.00 |
| MAPKAPK5-AS1 | 0.91 | 0.83 | 1.00 | 0.05 |
| AC104389.2 | 30442.08 | 15.00 | 61774747.19 | 0.01 |
| PDE9A | 1.07 | 1.00 | 1.15 | 0.04 |
| AADAC | 1.01 | 1.00 | 1.02 | 0.05 |
| FRMD6 | 1.09 | 1.04 | 1.15 | 0.00 |
| MIR3119-2 | 6.84 | 1.09 | 42.67 | 0.04 |
| COL10A1 | 1.01 | 1.00 | 1.02 | 0.03 |
| PMS2P10 | 0.39 | 0.17 | 0.88 | 0.02 |
| FAM81A | 0.83 | 0.72 | 0.95 | 0.01 |
| DPP3 | 0.97 | 0.95 | 1.00 | 0.02 |
| AC010719.1 | 0.81 | 0.68 | 0.97 | 0.02 |
| LINC02523 | 11205.64 | 18.65 | 6731074.91 | 0.00 |
| RASA4DP | 1.44 | 1.04 | 2.01 | 0.03 |
| AC120045.3 | 81.94 | 1.67 | 4029.11 | 0.03 |
| NPC2 | 1.01 | 1.00 | 1.02 | 0.00 |
| OR2M2 | 18.85 | 1.21 | 292.68 | 0.04 |
| CCDC80 | 1.01 | 1.00 | 1.02 | 0.03 |
| DEFB113 | 9.34 | 1.91 | 45.80 | 0.01 |
| PNPLA6 | 0.95 | 0.91 | 1.00 | 0.04 |
| LUM | 1.00 | 1.00 | 1.00 | 0.01 |
| CLRN3 | 0.99 | 0.97 | 1.00 | 0.02 |
| RAC1P9 | 9.19 | 1.27 | 66.70 | 0.03 |
| MTCYBP5 | 21.77 | 1.33 | 356.42 | 0.03 |
| CD82 | 0.99 | 0.97 | 1.00 | 0.02 |
| AC079347.1 | 6.02 | 1.15 | 31.59 | 0.03 |
| AL354761.1 | 14.37 | 1.97 | 104.94 | 0.01 |
| AC093908.1 | 3.14 | 1.34 | 7.38 | 0.01 |
| AC012055.1 | 3.76 | 1.03 | 13.72 | 0.04 |
| SLC35A3 | 0.96 | 0.93 | 0.99 | 0.01 |
| AL139281.1 | 26.35 | 2.36 | 294.66 | 0.01 |
| AL138830.1 | 8.08 | 1.11 | 59.01 | 0.04 |
| CTAGE14P | 72.38 | 3.76 | 1391.66 | 0.00 |
| RPA2P1 | 534.67 | 13.51 | 21162.48 | 0.00 |
| YWHAEP2 | 21.25 | 1.10 | 409.41 | 0.04 |
| LGR6 | 0.96 | 0.92 | 0.99 | 0.01 |
| AC073359.1 | 20.57 | 2.17 | 194.66 | 0.01 |
| PGAM5P1 | 25624.71 | 20.53 | 31984964.51 | 0.01 |
| PDGFD | 1.06 | 1.01 | 1.11 | 0.03 |
| AC117500.3 | 92.02 | 2.93 | 2891.24 | 0.01 |
| IGHVII-15-1 | 9.97 | 1.27 | 78.46 | 0.03 |
| CACNA2D3 | 1.64 | 1.01 | 2.68 | 0.05 |
| RBMXP1 | 13.00 | 1.58 | 107.04 | 0.02 |
| AKAP12 | 1.02 | 1.00 | 1.03 | 0.02 |
| OR10A5 | 15.68 | 2.56 | 95.92 | 0.00 |
| FAM81B | 1.45 | 1.05 | 2.01 | 0.02 |
| TRPC3 | 1.59 | 1.02 | 2.48 | 0.04 |
| AL445183.2 | 2.30 | 1.23 | 4.29 | 0.01 |
| OR6N1 | 417.81 | 2.00 | 87243.60 | 0.03 |
| FLRT2 | 1.38 | 1.14 | 1.66 | 0.00 |
| RNA5SP408 | 7.21 | 1.85 | 28.07 | 0.00 |
| FAM83H | 0.99 | 0.99 | 1.00 | 0.01 |
| FUT2 | 0.98 | 0.97 | 1.00 | 0.01 |
| AMHR2 | 1.11 | 1.04 | 1.19 | 0.00 |
| AL512649.1 | 11.68 | 2.24 | 60.97 | 0.00 |
| AL355922.1 | 1.28 | 1.04 | 1.58 | 0.02 |
| CDH2 | 1.04 | 1.02 | 1.07 | 0.00 |
| TIMM9P3 | 6.81 | 1.45 | 31.94 | 0.02 |
| TREML4 | 32.93 | 3.80 | 285.59 | 0.00 |
| AC022031.1 | 51.13 | 4.87 | 536.67 | 0.00 |
| DCN | 1.01 | 1.00 | 1.01 | 0.03 |
| MEMO1P5 | 4.96 | 1.21 | 20.40 | 0.03 |
| CFAP157 | 0.63 | 0.42 | 0.95 | 0.03 |
| RNA5SP499 | 57.81 | 6.56 | 509.42 | 0.00 |
| AC009163.5 | 0.00 | 0.00 | 0.04 | 0.01 |
| CLSPN | 0.89 | 0.80 | 0.97 | 0.01 |
| C19orf71 | 0.89 | 0.83 | 0.96 | 0.00 |
| AC006328.1 | 320.75 | 1.59 | 64513.16 | 0.03 |
| YWHABP2 | 1.65 | 1.24 | 2.19 | 0.00 |
| KBTBD2 | 1.05 | 1.01 | 1.10 | 0.03 |
| ALDH5A1 | 0.95 | 0.91 | 0.99 | 0.02 |
| LEF1-AS1 | 6.45 | 1.28 | 32.33 | 0.02 |
| PPM1J | 0.47 | 0.27 | 0.83 | 0.01 |
| PAK3 | 1.10 | 1.01 | 1.19 | 0.04 |
| SNORD115-33 | 13.28 | 1.86 | 94.80 | 0.01 |
| PLCL1 | 1.53 | 1.20 | 1.96 | 0.00 |
| ZNF736P4Y | 41635.70 | 1.63 | 1065098577.00 | 0.04 |
| OLIG3 | 1.89 | 1.04 | 3.45 | 0.04 |
| AL008638.1 | 7.50 | 1.48 | 38.10 | 0.02 |
| TMEM233 | 2.10 | 1.11 | 3.98 | 0.02 |
| RAB11FIP4 | 0.88 | 0.80 | 0.98 | 0.02 |
| RN7SL841P | 83.76 | 2.77 | 2534.60 | 0.01 |
| DNM2 | 0.98 | 0.96 | 1.00 | 0.05 |
| AC023078.1 | 74.53 | 4.68 | 1186.82 | 0.00 |
| AL161713.1 | 5.19 | 1.04 | 26.01 | 0.05 |
| PABPC1P2 | 7.47 | 1.47 | 38.09 | 0.02 |
| BEX4 | 1.02 | 1.00 | 1.03 | 0.04 |
| VIM | 1.00 | 1.00 | 1.01 | 0.00 |
| AC008687.4 | 1.24 | 1.04 | 1.49 | 0.02 |
| TECTA | 7.83 | 1.71 | 35.87 | 0.01 |
| TRBV22-1 | 4.67 | 1.20 | 18.17 | 0.03 |
| PRDM5 | 1.59 | 1.04 | 2.44 | 0.03 |
| AC010374.1 | 74.08 | 2.54 | 2162.06 | 0.01 |
| RNF217 | 1.24 | 1.04 | 1.49 | 0.02 |
| MIR8056 | 12.41 | 1.09 | 140.81 | 0.04 |
| GCDH | 0.86 | 0.75 | 0.98 | 0.03 |
| AL353752.1 | 1.93 | 1.25 | 2.99 | 0.00 |
| AL357274.1 | 2.30 | 1.16 | 4.53 | 0.02 |
| AC087627.1 | 6.99 | 1.55 | 31.54 | 0.01 |
| MIR5011 | 2.64 | 1.14 | 6.09 | 0.02 |
| AC010608.3 | 1297.59 | 25.91 | 64973.03 | 0.00 |
| HERC2P3 | 1.25 | 1.00 | 1.54 | 0.05 |
| AC093627.1 | 1.70 | 1.29 | 2.23 | 0.00 |
| MIR6862-1 | 144.60 | 7.37 | 2838.58 | 0.00 |
| CRTAC1 | 1.15 | 1.03 | 1.30 | 0.02 |
| FZD7 | 1.01 | 1.00 | 1.02 | 0.03 |
| BTG4P1 | 3.46 | 1.07 | 11.22 | 0.04 |
| AC114757.1 | 20.72 | 3.00 | 143.24 | 0.00 |
| AC087360.1 | 110.36 | 6.35 | 1916.62 | 0.00 |
| AP001117.1 | 9.56 | 1.59 | 57.65 | 0.01 |
| OTOS | 6.07 | 1.40 | 26.29 | 0.02 |
| PRPF3 | 0.93 | 0.86 | 0.99 | 0.03 |
| AL391832.3 | 4.56 | 1.56 | 13.32 | 0.01 |
| AC094104.2 | 13.50 | 2.42 | 75.17 | 0.00 |
| AL589994.2 | 60.09 | 4.24 | 851.76 | 0.00 |
| BICDL1 | 0.92 | 0.86 | 0.99 | 0.02 |
| KCNK2 | 1.27 | 1.07 | 1.52 | 0.01 |
| CD109 | 1.04 | 1.02 | 1.07 | 0.00 |
| LINC01315 | 0.78 | 0.66 | 0.92 | 0.00 |
| AL445218.1 | 21.21 | 1.39 | 324.59 | 0.03 |
| AC020699.1 | 832.28 | 2.72 | 254587.82 | 0.02 |
| AC005586.1 | 0.77 | 0.65 | 0.90 | 0.00 |
| AC021549.1 | 2.03 | 1.14 | 3.61 | 0.02 |
| OR13G1 | 148.85 | 2.36 | 9389.30 | 0.02 |
| CACTIN | 0.81 | 0.70 | 0.95 | 0.01 |
| TM4SF18 | 1.07 | 1.00 | 1.15 | 0.04 |
| AC118282.4 | 18.62 | 3.00 | 115.55 | 0.00 |
| AC006355.2 | 5.09 | 1.38 | 18.82 | 0.01 |
| AC068044.1 | 82.77 | 2.24 | 3058.39 | 0.02 |
| PAPSS2 | 1.02 | 1.00 | 1.04 | 0.04 |
| Z99129.4 | 1.08 | 1.00 | 1.16 | 0.04 |
| AP002989.1 | 30.32 | 3.63 | 253.54 | 0.00 |
| COL8A1 | 1.02 | 1.00 | 1.03 | 0.02 |
| LINC01925 | 1.47 | 1.11 | 1.95 | 0.01 |
| AC023141.9 | 1904.44 | 2.30 | 1575184.47 | 0.03 |
| ISY1 | 0.84 | 0.73 | 0.98 | 0.03 |
| OR2L9P | 146.05 | 3.44 | 6195.12 | 0.01 |
| GXYLT1P2 | 25.97 | 1.79 | 377.40 | 0.02 |
| AC009227.2 | 7.40 | 1.26 | 43.53 | 0.03 |
| SNRPGP13 | 2.62 | 1.17 | 5.86 | 0.02 |
| COX15 | 0.95 | 0.90 | 0.99 | 0.02 |
| OR10G5P | 8.57 | 1.14 | 64.30 | 0.04 |
| IGLVI-68 | 4.14 | 1.25 | 13.70 | 0.02 |
| MPND | 0.93 | 0.88 | 0.99 | 0.02 |
| AC087241.1 | 29.46 | 3.37 | 257.57 | 0.00 |
| AC092574.1 | 0.69 | 0.49 | 0.97 | 0.03 |
| AC002094.4 | 1.44 | 1.03 | 2.00 | 0.03 |
| GPX8 | 1.05 | 1.02 | 1.08 | 0.00 |
| LMX1A-AS2 | 5.30 | 1.49 | 18.87 | 0.01 |
| AC073130.1 | 19.80 | 1.80 | 217.76 | 0.01 |
| AP005273.1 | 38.99 | 3.03 | 502.20 | 0.00 |
| MIR654 | 19.06 | 2.73 | 132.91 | 0.00 |
| CCDC54 | 112.65 | 8.18 | 1550.54 | 0.00 |
| NRXN2-AS1 | 46.37 | 3.81 | 564.44 | 0.00 |
| AC126467.1 | 22.17 | 5.41 | 90.87 | 0.00 |
| LINC00388 | 138.00 | 1.51 | 12589.41 | 0.03 |
| UBL7-AS1 | 0.59 | 0.40 | 0.87 | 0.01 |
| SERPINE1 | 1.00 | 1.00 | 1.00 | 0.00 |
| LINC02529 | 44.06 | 2.85 | 682.26 | 0.01 |
| FAAH | 0.92 | 0.88 | 0.97 | 0.00 |
| ZNF521 | 1.18 | 1.07 | 1.30 | 0.00 |
| AC010546.1 | 28.31 | 3.06 | 261.91 | 0.00 |
| AC007225.2 | 7.51 | 1.31 | 43.01 | 0.02 |
| HNRNPH3P1 | 14.79 | 1.08 | 202.90 | 0.04 |
| AL160408.4 | 2.94 | 1.66 | 5.22 | 0.00 |
| IER5L | 0.99 | 0.97 | 1.00 | 0.04 |
| NTRK3 | 4.39 | 1.09 | 17.60 | 0.04 |
| AL445437.1 | 1.81 | 1.31 | 2.51 | 0.00 |
| SEC23A | 1.05 | 1.01 | 1.08 | 0.01 |
| GAPDHP29 | 40.63 | 3.17 | 521.51 | 0.00 |
| FGF1 | 2.21 | 1.40 | 3.49 | 0.00 |
| THOP1 | 0.92 | 0.87 | 0.98 | 0.01 |
| C5orf66-AS2 | 73.27 | 2.52 | 2134.75 | 0.01 |
| AC069224.1 | 9.53 | 2.40 | 37.76 | 0.00 |
| KCND2 | 2.04 | 1.30 | 3.19 | 0.00 |
| GHR | 1.19 | 1.05 | 1.35 | 0.01 |
| OR5D16 | 81.34 | 1.41 | 4690.45 | 0.03 |
| ARL2BPP2 | 235.23 | 2.26 | 24475.46 | 0.02 |
| RNU6-394P | 589.35 | 1.95 | 177742.15 | 0.03 |
| AL353612.1 | 15.26 | 2.11 | 110.37 | 0.01 |
| BX510359.4 | 12.47 | 1.63 | 95.52 | 0.02 |
| LINC01497 | 2.61 | 1.13 | 5.99 | 0.02 |
| AC022467.1 | 11.28 | 2.25 | 56.64 | 0.00 |
| MAP7 | 0.97 | 0.95 | 1.00 | 0.04 |
| RPL23AP68 | 5.53 | 1.12 | 27.33 | 0.04 |
| TRGJP | 15.22 | 4.36 | 53.13 | 0.00 |
| AL596268.1 | 35106.46 | 46.65 | 26417239.94 | 0.00 |
| DCBLD2 | 1.05 | 1.01 | 1.10 | 0.03 |
| PSAT1 | 0.99 | 0.98 | 1.00 | 0.05 |
| LRRC70 | 2.51 | 1.30 | 4.87 | 0.01 |
| AL627095.1 | 4.16 | 1.25 | 13.84 | 0.02 |
| AC104619.3 | 1.36 | 1.06 | 1.74 | 0.02 |
| MCC | 1.16 | 1.06 | 1.27 | 0.00 |
| AC079178.1 | 92384.70 | 314.44 | 27143155.91 | 0.00 |
| BMP4 | 0.98 | 0.97 | 1.00 | 0.01 |
| STEAP4 | 1.06 | 1.01 | 1.12 | 0.02 |
| PCOLCE2 | 1.15 | 1.01 | 1.31 | 0.03 |
| AL365396.1 | 30.41 | 1.24 | 745.88 | 0.04 |
| RNU6-276P | 3.37 | 1.18 | 9.60 | 0.02 |
| MIR199A2 | 1.68 | 1.02 | 2.76 | 0.04 |
| ASPA | 1.42 | 1.02 | 2.00 | 0.04 |
| AC074011.1 | 1.51 | 1.05 | 2.17 | 0.03 |
| RAI14 | 1.03 | 1.00 | 1.05 | 0.05 |
| PKNOX2 | 1.42 | 1.01 | 1.99 | 0.05 |
| STIL | 0.93 | 0.87 | 0.99 | 0.03 |
| RNA5SP164 | 12.56 | 2.27 | 69.44 | 0.00 |
| IGHV3-25 | 1.12 | 1.01 | 1.23 | 0.03 |
| MIR381HG | 7.24 | 1.72 | 30.50 | 0.01 |
| AC117482.1 | 75.41 | 2.67 | 2130.73 | 0.01 |
| AP002353.1 | 78.63 | 5.01 | 1233.07 | 0.00 |
| PTH1R | 1.43 | 1.06 | 1.93 | 0.02 |
| GSR | 0.99 | 0.98 | 1.00 | 0.03 |
| AL157371.1 | 10.47 | 2.17 | 50.59 | 0.00 |
| CMTM3 | 1.02 | 1.00 | 1.05 | 0.04 |
| LHFPL6 | 1.01 | 1.00 | 1.02 | 0.02 |
| HIGD1B | 1.68 | 1.25 | 2.26 | 0.00 |
| CALCR | 1.23 | 1.01 | 1.51 | 0.04 |
| AK1 | 0.90 | 0.83 | 0.97 | 0.01 |
| SUDS3 | 0.93 | 0.88 | 0.99 | 0.03 |
| LINC02115 | 3.13 | 1.25 | 7.80 | 0.01 |
| BATF2 | 0.97 | 0.95 | 0.99 | 0.01 |
| AC131211.1 | 12.98 | 2.39 | 70.51 | 0.00 |
| AC027369.5 | 18.56 | 2.03 | 169.34 | 0.01 |
| COLEC12 | 1.14 | 1.06 | 1.23 | 0.00 |
| AL807761.4 | 29.67 | 1.02 | 863.65 | 0.05 |
| ETV6 | 0.95 | 0.91 | 1.00 | 0.03 |
| SNORD113-5 | 5.00 | 1.73 | 14.46 | 0.00 |
| MDFIC | 1.05 | 1.00 | 1.09 | 0.04 |
| RDH13 | 0.87 | 0.76 | 0.99 | 0.03 |
| MYH14 | 0.99 | 0.99 | 1.00 | 0.02 |
| CUL4A | 0.96 | 0.93 | 1.00 | 0.05 |
| PPP1R1C | 1.49 | 1.15 | 1.93 | 0.00 |
| CPNE8-AS1 | 1.92 | 1.08 | 3.41 | 0.03 |
| AC010768.2 | 0.66 | 0.45 | 0.97 | 0.04 |
| RGS2 | 1.01 | 1.00 | 1.02 | 0.00 |
| VPS35L | 1.12 | 1.03 | 1.23 | 0.01 |
| TNNI3K | 62.25 | 1.55 | 2498.58 | 0.03 |
| AL355095.1 | 16.37 | 2.37 | 112.99 | 0.00 |
| AC110015.1 | 1.76 | 1.04 | 2.99 | 0.03 |
| MIR5094 | 0.84 | 0.73 | 0.97 | 0.02 |
| AC012186.3 | 3.34 | 1.15 | 9.74 | 0.03 |
| ARMCX2 | 1.06 | 1.02 | 1.11 | 0.01 |
| PTPRQ | 8.15 | 1.65 | 40.14 | 0.01 |
| AL713866.2 | 18.19 | 2.21 | 149.49 | 0.01 |
| FAM111A | 0.93 | 0.87 | 0.99 | 0.02 |
| AC025253.1 | 7.01 | 1.54 | 31.82 | 0.01 |
| AP006587.5 | 59.16 | 2.49 | 1404.28 | 0.01 |
| SERPINA5 | 1.02 | 1.00 | 1.03 | 0.01 |
| LINC02353 | 14.42 | 1.86 | 111.94 | 0.01 |
| GGT5 | 1.02 | 1.01 | 1.04 | 0.00 |
| AL139135.2 | 20.83 | 2.81 | 154.65 | 0.00 |
| CC2D2A | 1.19 | 1.04 | 1.36 | 0.01 |
| LBH | 1.02 | 1.01 | 1.04 | 0.00 |
| AC112203.1 | 16.08 | 1.77 | 146.26 | 0.01 |
| MRPS21P5 | 16.34 | 2.25 | 118.76 | 0.01 |
| MTND5P41 | 14.96 | 1.45 | 154.67 | 0.02 |
| AC006482.1 | 4.92 | 1.59 | 15.16 | 0.01 |
| TTC9C | 0.89 | 0.82 | 0.98 | 0.01 |
| SNORD116-22 | 3.43 | 1.15 | 10.22 | 0.03 |
| AC097484.1 | 3.21 | 1.01 | 10.25 | 0.05 |
| NTAN1P3 | 4.27 | 1.14 | 15.98 | 0.03 |
| AC004882.1 | 0.39 | 0.17 | 0.90 | 0.03 |
| APLP2 | 1.01 | 1.00 | 1.01 | 0.03 |
| CCPG1 | 1.23 | 1.06 | 1.43 | 0.01 |
| SNORA2B | 0.66 | 0.47 | 0.94 | 0.02 |
| AL132822.1 | 4.88 | 2.28 | 10.49 | 0.00 |
| AC091705.1 | 2.20 | 1.31 | 3.67 | 0.00 |
| MIR217HG | 8.78 | 2.02 | 38.15 | 0.00 |
| OR10G4 | 272.06 | 4.93 | 15005.10 | 0.01 |
| RNU6-1260P | 27.00 | 2.95 | 246.80 | 0.00 |
| FAM43B | 1.74 | 1.09 | 2.78 | 0.02 |
| AC019055.1 | 40.41 | 2.30 | 709.81 | 0.01 |
| BLOC1S2P1 | 27.66 | 1.50 | 510.50 | 0.03 |
| AL132709.4 | 8.15 | 2.61 | 25.43 | 0.00 |
| HTR1F | 20.43 | 2.47 | 169.04 | 0.01 |
| LINC02730 | 62.83 | 4.83 | 817.20 | 0.00 |
| AL049833.1 | 3.61 | 1.05 | 12.37 | 0.04 |
| MED8-AS1 | 0.52 | 0.28 | 0.97 | 0.04 |
| AC009063.2 | 5.41 | 1.05 | 27.97 | 0.04 |
| LIN7A | 1.21 | 1.05 | 1.39 | 0.01 |
| AC092925.1 | 7.21 | 1.64 | 31.70 | 0.01 |
| ZNF282 | 0.94 | 0.90 | 0.98 | 0.01 |
| GNAI1 | 1.06 | 1.01 | 1.11 | 0.02 |
| CYB561D1 | 0.84 | 0.72 | 0.98 | 0.02 |
| TSHZ3 | 1.05 | 1.00 | 1.10 | 0.04 |
| GRAMD4P7 | 11.38 | 1.11 | 117.19 | 0.04 |
| GEMIN8P3 | 265.97 | 4.20 | 16828.91 | 0.01 |
| AC107958.1 | 804.96 | 2.33 | 278494.14 | 0.02 |
| TRGVA | 7.98 | 2.20 | 28.98 | 0.00 |
| LINC02260 | 30.51 | 1.66 | 561.03 | 0.02 |
| SLITRK2 | 2.27 | 1.02 | 5.05 | 0.05 |
| KLHL41 | 1.25 | 1.02 | 1.55 | 0.03 |
| TYK2 | 0.95 | 0.90 | 0.99 | 0.01 |
| RUNX2 | 1.10 | 1.00 | 1.20 | 0.04 |
| ANXA5 | 1.00 | 1.00 | 1.01 | 0.00 |
| AL132855.1 | 28.21 | 1.75 | 454.80 | 0.02 |
| SLC27A2 | 0.96 | 0.92 | 1.00 | 0.03 |
| MISP3 | 0.96 | 0.93 | 0.99 | 0.00 |
| AC027688.1 | 8.13 | 1.39 | 47.48 | 0.02 |
| SPATA2P1 | 21.82 | 1.02 | 468.90 | 0.05 |
| AC098934.3 | 1.06 | 1.00 | 1.13 | 0.04 |
| ZNF557 | 0.73 | 0.56 | 0.94 | 0.01 |
| CGB3 | 1.05 | 1.00 | 1.10 | 0.03 |
| AC131055.1 | 34.04 | 2.70 | 429.82 | 0.01 |
| AC123904.3 | 3.90 | 1.10 | 13.83 | 0.03 |
| RPS6KA1 | 0.97 | 0.94 | 0.99 | 0.01 |
| AC090204.1 | 1.04 | 1.00 | 1.08 | 0.05 |
| RNU6-309P | 3.98 | 1.41 | 11.26 | 0.01 |
| AL929410.1 | 10.08 | 1.52 | 66.75 | 0.02 |
| KIFAP3 | 1.07 | 1.01 | 1.14 | 0.02 |
| H2BP5 | 36.33 | 1.72 | 767.40 | 0.02 |
| SMARCA1 | 1.03 | 1.00 | 1.06 | 0.04 |
| HLX-AS1 | 6.74 | 1.78 | 25.57 | 0.01 |
| SEPTIN4 | 1.39 | 1.10 | 1.76 | 0.01 |
| UGT2B24P | 51.69 | 3.09 | 863.69 | 0.01 |
| BOLA2 | 851.40 | 18.43 | 39331.43 | 0.00 |
| MIR621 | 0.97 | 0.95 | 1.00 | 0.02 |
| DDIAS | 0.87 | 0.76 | 0.98 | 0.03 |
| FCHO1 | 0.92 | 0.86 | 0.99 | 0.03 |
| PDZPH1P | 24.57 | 5.03 | 120.06 | 0.00 |
| ARHGAP44 | 0.88 | 0.79 | 0.99 | 0.03 |
| MAN1C1 | 1.13 | 1.03 | 1.23 | 0.01 |
| HTRA3 | 1.01 | 1.00 | 1.02 | 0.01 |
| AC095041.1 | 2.33 | 1.31 | 4.16 | 0.00 |
| FKBP14 | 1.14 | 1.02 | 1.26 | 0.02 |
| AL354861.3 | 5.92 | 1.26 | 27.93 | 0.02 |
| ECM2 | 1.10 | 1.03 | 1.18 | 0.00 |
| RNU6-544P | 8.50 | 1.27 | 56.96 | 0.03 |
| SURF6 | 0.95 | 0.91 | 1.00 | 0.04 |
| AC099786.3 | 111.32 | 3.15 | 3932.58 | 0.01 |
| FAT3 | 1.41 | 1.01 | 1.98 | 0.04 |
| AL157817.1 | 97.76 | 4.03 | 2372.12 | 0.00 |
| AC010203.1 | 0.50 | 0.32 | 0.80 | 0.00 |
| NDUFS5P6 | 6.08 | 1.05 | 35.14 | 0.04 |
| TMEM210 | 1.83 | 1.19 | 2.83 | 0.01 |
| CDC25A | 0.90 | 0.82 | 0.98 | 0.02 |
| ARAFP1 | 142.35 | 3.85 | 5260.21 | 0.01 |
| TRAJ59 | 5.44 | 2.31 | 12.84 | 0.00 |
| PAPPA | 1.04 | 1.00 | 1.07 | 0.05 |
| AC103996.2 | 3.86 | 1.62 | 9.18 | 0.00 |
| RARRES2P9 | 4.88 | 1.20 | 19.76 | 0.03 |
| RNU7-66P | 10.11 | 2.04 | 50.08 | 0.00 |
| PTPRD | 1.30 | 1.11 | 1.52 | 0.00 |
| MCM3 | 0.99 | 0.98 | 1.00 | 0.02 |
| HAGLR | 1.08 | 1.02 | 1.15 | 0.01 |
| TPPP2 | 12.24 | 1.11 | 135.06 | 0.04 |
| TUBAP | 15.36 | 1.07 | 219.63 | 0.04 |
| ADGRF4 | 1.10 | 1.02 | 1.19 | 0.01 |
| PIDD1 | 0.88 | 0.78 | 0.99 | 0.03 |
| LINC01698 | 7.64 | 1.20 | 48.73 | 0.03 |
| RNU6-148P | 2.45 | 1.09 | 5.53 | 0.03 |
| GMCL1 | 0.93 | 0.88 | 0.98 | 0.01 |
| DPH3P2 | 179.68 | 2.63 | 12266.76 | 0.02 |
| ASF1B | 0.97 | 0.95 | 0.99 | 0.01 |
| FECHP1 | 23.95 | 1.62 | 354.33 | 0.02 |
| SLC13A5 | 1.07 | 1.01 | 1.14 | 0.03 |
| AL109946.1 | 47.71 | 2.74 | 830.64 | 0.01 |
| OSBPL9 | 1.09 | 1.03 | 1.15 | 0.00 |
| SOD2P1 | 9.21 | 1.44 | 58.98 | 0.02 |
| AC107021.2 | 5.38 | 2.14 | 13.55 | 0.00 |
| WWP1P1 | 12.50 | 1.96 | 79.73 | 0.01 |
| CXCR4 | 1.00 | 1.00 | 1.01 | 0.01 |
| PDGFRL | 1.08 | 1.04 | 1.12 | 0.00 |
| COL4A5 | 1.04 | 1.00 | 1.08 | 0.04 |
| DDX43P1 | 276.15 | 2.46 | 31012.70 | 0.02 |
| AC245505.1 | 1078.49 | 5.12 | 227320.03 | 0.01 |
| MIR329-2 | 7.54 | 1.30 | 43.67 | 0.02 |
| RN7SKP293 | 59.63 | 3.40 | 1045.77 | 0.01 |
| LMNTD2 | 0.82 | 0.68 | 0.98 | 0.03 |
| VWA8P1 | 92.30 | 7.94 | 1072.78 | 0.00 |
| NOS1AP | 0.70 | 0.50 | 0.97 | 0.03 |
| AC134312.6 | 3.95 | 1.11 | 14.09 | 0.03 |
| ZNF885P | 499.32 | 1.88 | 132492.34 | 0.03 |
| OR11H3P | 2666380.96 | 172.41 | 41235390248.00 | 0.00 |
| CSRNP1 | 1.02 | 1.00 | 1.03 | 0.04 |
| AC104090.1 | 24.49 | 1.29 | 463.11 | 0.03 |
| SRMS | 0.95 | 0.90 | 0.99 | 0.03 |
| GLI1 | 1.12 | 1.02 | 1.23 | 0.02 |
| AC022418.1 | 85.09 | 1.44 | 5035.92 | 0.03 |
| OR51A6P | 325.98 | 17.43 | 6095.75 | 0.00 |
| CSK | 0.98 | 0.96 | 1.00 | 0.04 |
| MIR767 | 29.47 | 3.86 | 224.69 | 0.00 |
| RPS29P9 | 1.42 | 1.02 | 1.99 | 0.04 |
| KIF24 | 0.83 | 0.72 | 0.95 | 0.01 |
| LINC01815 | 7.44 | 1.91 | 28.90 | 0.00 |
| ADAMTS1 | 1.02 | 1.01 | 1.04 | 0.00 |
| CYMP-AS1 | 2.41 | 1.68 | 3.46 | 0.00 |
| RNU2-20P | 10.51 | 1.69 | 65.36 | 0.01 |
| MIR1298 | 2.71 | 1.18 | 6.24 | 0.02 |
| LAMA2 | 1.07 | 1.03 | 1.12 | 0.00 |
| RNU6-1220P | 3.84 | 1.11 | 13.27 | 0.03 |
| TMEM259 | 0.97 | 0.95 | 0.99 | 0.01 |
| DAZ2 | 2.86 | 1.14 | 7.19 | 0.03 |
| DPP4-DT | 4.11 | 1.15 | 14.64 | 0.03 |
| C9orf64 | 0.94 | 0.89 | 0.99 | 0.03 |
| NFE4 | 1.25 | 1.10 | 1.43 | 0.00 |
| OR2W5 | 25.51 | 1.53 | 423.86 | 0.02 |
| QKI | 1.12 | 1.05 | 1.20 | 0.00 |
| HDAC8 | 0.75 | 0.60 | 0.94 | 0.01 |
| AC105148.1 | 11.97 | 1.43 | 99.96 | 0.02 |
| LINC02454 | 1.31 | 1.13 | 1.51 | 0.00 |
| HMGB1P17 | 0.06 | 0.00 | 0.85 | 0.04 |
| AL359999.1 | 11.61 | 1.98 | 68.23 | 0.01 |
| AL049775.1 | 21.64 | 5.88 | 79.68 | 0.00 |
| RNU6-640P | 0.62 | 0.40 | 0.97 | 0.04 |
| RPL19P16 | 1.14 | 1.01 | 1.28 | 0.03 |
| AC236972.2 | 4.40 | 1.11 | 17.52 | 0.04 |
| AL356473.1 | 274.92 | 6.55 | 11531.53 | 0.00 |
| PCDHB4 | 1.24 | 1.02 | 1.51 | 0.03 |
| AJ239318.1 | 345.64 | 38.53 | 3100.34 | 0.00 |
| OR4A48P | 4.38 | 1.05 | 18.27 | 0.04 |
| AC005632.2 | 2.78 | 1.51 | 5.11 | 0.00 |
| ZNF965P | 75.02 | 1.85 | 3046.18 | 0.02 |
| AL359555.1 | 2.86 | 1.23 | 6.65 | 0.02 |
| EEF1A1P34 | 11.45 | 2.62 | 49.97 | 0.00 |
| TSSK6 | 0.73 | 0.56 | 0.96 | 0.02 |
| RPL23AP59 | 2.36 | 1.26 | 4.44 | 0.01 |
| AC004946.1 | 10.80 | 1.08 | 107.98 | 0.04 |
| MTND4LP20 | 282.69 | 13.11 | 6097.70 | 0.00 |
| EGFLAM-AS3 | 10.90 | 2.07 | 57.43 | 0.00 |
| MCRIP2P2 | 12.58 | 1.85 | 85.50 | 0.01 |
| RNU7-88P | 3.45 | 1.02 | 11.70 | 0.05 |
| LINC02295 | 1.94 | 1.10 | 3.42 | 0.02 |
| LINC00400 | 6.00 | 1.05 | 34.30 | 0.04 |
| MIR4528 | 7.00 | 1.64 | 29.84 | 0.01 |
| ZFYVE27 | 0.92 | 0.86 | 0.97 | 0.00 |
| RN7SKP116 | 1.99 | 1.01 | 3.90 | 0.05 |
| AP000920.1 | 6.35 | 1.47 | 27.36 | 0.01 |
| AC104623.1 | 28.08 | 2.89 | 273.18 | 0.00 |
| GALNT16 | 1.27 | 1.02 | 1.58 | 0.03 |
| AL137027.1 | 33.98 | 1.33 | 867.32 | 0.03 |
| VSX2 | 21.39 | 1.97 | 232.08 | 0.01 |
| MRPS18CP4 | 2.12 | 1.25 | 3.58 | 0.01 |
| IRAK1 | 0.99 | 0.98 | 1.00 | 0.01 |
| AC092850.1 | 1490949.56 | 16.81 | 132273000000.00 | 0.01 |
| SAMD1 | 0.98 | 0.97 | 0.99 | 0.01 |
| AC139099.3 | 5152.14 | 12.47 | 2128441.98 | 0.01 |
| DAB2 | 1.03 | 1.01 | 1.05 | 0.00 |
| RNA5SP461 | 2.57 | 1.13 | 5.84 | 0.02 |
| KRTAP8-1 | 4866.29 | 108.71 | 217831.18 | 0.00 |
| AC007391.1 | 20.72 | 3.88 | 110.71 | 0.00 |
| GNG11 | 1.05 | 1.02 | 1.08 | 0.00 |
| NUDT11 | 1.24 | 1.02 | 1.52 | 0.03 |
| LINC01896 | 42.52 | 1.47 | 1226.79 | 0.03 |
| DOT1L | 0.92 | 0.87 | 0.98 | 0.01 |
| AL353611.1 | 17.92 | 1.70 | 188.50 | 0.02 |
| AC136932.1 | 23.72 | 1.62 | 347.03 | 0.02 |
| NF1P12 | 6.68 | 1.34 | 33.45 | 0.02 |
| AC147067.2 | 1.38 | 1.08 | 1.76 | 0.01 |
| SPRED1 | 1.09 | 1.03 | 1.16 | 0.00 |
| BPI | 1.10 | 1.00 | 1.20 | 0.05 |
| ADAM6 | 41.90 | 1.09 | 1613.28 | 0.04 |
| CHROMR | 1.21 | 1.04 | 1.42 | 0.01 |
| AC006065.5 | 621.85 | 8.22 | 47059.19 | 0.00 |
| OR52X1P | 48.12 | 4.07 | 569.20 | 0.00 |
| AC013652.1 | 1.51 | 1.00 | 2.26 | 0.05 |
| AC010451.3 | 5.58 | 1.29 | 24.16 | 0.02 |
| RMI1 | 0.90 | 0.84 | 0.98 | 0.01 |
| AC244153.1 | 1.11 | 1.01 | 1.23 | 0.03 |
| ADAM24P | 43.54 | 1.61 | 1174.13 | 0.02 |
| TFPI2 | 1.01 | 1.00 | 1.02 | 0.01 |
| CNTNAP3P1 | 689.37 | 5.61 | 84739.41 | 0.01 |
| AC145141.1 | 12.25 | 2.08 | 72.21 | 0.01 |
| RIT2 | 14.09 | 3.91 | 50.76 | 0.00 |
| ZNF823 | 0.86 | 0.75 | 0.98 | 0.03 |
| PPAN | 0.82 | 0.68 | 0.98 | 0.03 |
| AL139406.1 | 4.87 | 1.33 | 17.89 | 0.02 |
| FAM111B | 0.94 | 0.89 | 1.00 | 0.04 |
| FMOD | 1.00 | 1.00 | 1.00 | 0.04 |
| AC008164.1 | 4762.38 | 25.46 | 890660.26 | 0.00 |
| AC009784.1 | 8.62 | 1.46 | 50.84 | 0.02 |
| MIR1305 | 8.76 | 1.51 | 50.76 | 0.02 |
| UBE2QL1 | 1.29 | 1.05 | 1.57 | 0.01 |
| MED18 | 0.87 | 0.80 | 0.94 | 0.00 |
| AC008013.1 | 1.91 | 1.04 | 3.53 | 0.04 |
| NEK5 | 0.49 | 0.29 | 0.83 | 0.01 |
| AGBL3 | 2.88 | 1.02 | 8.09 | 0.05 |
| LINC00968 | 2.36 | 1.11 | 5.03 | 0.03 |
| BX322635.1 | 2.55 | 1.11 | 5.87 | 0.03 |
| IL17RA | 0.90 | 0.82 | 0.99 | 0.03 |
| BRIP1 | 0.78 | 0.64 | 0.95 | 0.01 |
| CDH6 | 1.30 | 1.11 | 1.51 | 0.00 |
| SPESP1 | 1.08 | 1.01 | 1.15 | 0.02 |
| AKR1B1 | 1.03 | 1.01 | 1.04 | 0.00 |
| GPR50-AS1 | 1117.12 | 5.92 | 210685.00 | 0.01 |
| LINC02657 | 1.09 | 1.04 | 1.14 | 0.00 |
| AL365222.1 | 2.18 | 1.11 | 4.30 | 0.02 |
| PCDH7 | 1.03 | 1.00 | 1.06 | 0.03 |
| CNTN4-AS1 | 234.42 | 4.45 | 12339.34 | 0.01 |
| TSPAN7 | 1.02 | 1.00 | 1.03 | 0.04 |
| IGKV2-23 | 26.01 | 3.23 | 209.32 | 0.00 |
| FAM114A2 | 1.37 | 1.10 | 1.69 | 0.00 |
| AC010524.1 | 2.18 | 1.06 | 4.47 | 0.03 |
| ELOVL4 | 1.11 | 1.02 | 1.21 | 0.01 |
| AC110620.1 | 289.63 | 9.75 | 8599.57 | 0.00 |
| NECAB2 | 1.83 | 1.03 | 3.26 | 0.04 |
| AP000695.1 | 1.35 | 1.14 | 1.60 | 0.00 |
| AC080069.1 | 0.25 | 0.08 | 0.81 | 0.02 |
| AC009901.2 | 10.29 | 2.24 | 47.24 | 0.00 |
| AP003419.1 | 5.88 | 1.61 | 21.49 | 0.01 |
| AFAP1L1 | 1.16 | 1.05 | 1.28 | 0.00 |
| TCEAL7 | 1.09 | 1.01 | 1.18 | 0.02 |
| ABCA6 | 1.74 | 1.25 | 2.44 | 0.00 |
| TPRN | 0.96 | 0.93 | 1.00 | 0.03 |
| AC104758.1 | 3.54 | 1.56 | 8.02 | 0.00 |
| AL080273.1 | 6.60 | 1.44 | 30.30 | 0.02 |
| AC111000.6 | 8.69 | 1.60 | 47.18 | 0.01 |
| ADARB1 | 1.10 | 1.01 | 1.19 | 0.02 |
| LINC00106 | 0.87 | 0.77 | 0.99 | 0.03 |
| GUCY1A2 | 1.60 | 1.21 | 2.11 | 0.00 |
| OR51H1 | 63.26 | 4.31 | 928.32 | 0.00 |
| IMPACT | 1.07 | 1.02 | 1.11 | 0.00 |
| LINC02240 | 6.38 | 1.08 | 37.70 | 0.04 |
| AC004941.2 | 2.35 | 1.34 | 4.14 | 0.00 |
| MAP2K2 | 0.97 | 0.95 | 1.00 | 0.02 |
| PJA2 | 1.02 | 1.01 | 1.03 | 0.01 |
| SH3YL1 | 0.92 | 0.86 | 0.99 | 0.03 |
| IGKV2-38 | 4277.85 | 1.33 | 13782286.99 | 0.04 |
| POLQ | 0.85 | 0.73 | 0.98 | 0.03 |
| CRY2 | 1.08 | 1.02 | 1.14 | 0.01 |
| CDO1 | 1.16 | 1.03 | 1.31 | 0.01 |
| CD59 | 1.01 | 1.00 | 1.01 | 0.00 |
| OR6K5P | 9.67 | 1.06 | 88.02 | 0.04 |
| GALNT15 | 1.08 | 1.01 | 1.16 | 0.03 |
| CEP295 | 0.82 | 0.69 | 0.97 | 0.02 |
| AC009065.5 | 0.53 | 0.29 | 0.96 | 0.04 |
| OR4A4P | 388.04 | 7.57 | 19893.83 | 0.00 |
| AC005479.1 | 1.93 | 1.07 | 3.49 | 0.03 |
| CNTN4-AS2 | 6.91 | 1.26 | 37.97 | 0.03 |
| AC044873.1 | 5.85 | 1.19 | 28.63 | 0.03 |
| EIF4EP4 | 5.32 | 1.21 | 23.47 | 0.03 |
| LINC02186 | 5.53 | 1.25 | 24.43 | 0.02 |
| TRIM60P15 | 122.84 | 1.12 | 13431.41 | 0.04 |
| OR7E97P | 384.26 | 7.10 | 20788.19 | 0.00 |
| AP004833.3 | 26.86 | 1.48 | 486.18 | 0.03 |
| LINC00951 | 13.27 | 2.77 | 63.60 | 0.00 |
| AC068483.1 | 11.76 | 1.61 | 86.11 | 0.02 |
| GTF2F1 | 0.97 | 0.95 | 1.00 | 0.03 |
| CCNO | 0.94 | 0.89 | 0.99 | 0.03 |
| COG6 | 0.89 | 0.81 | 0.99 | 0.03 |
| UHRF1 | 0.92 | 0.87 | 0.98 | 0.00 |
| PABPC5 | 2.19 | 1.25 | 3.86 | 0.01 |
| DYNC1I1 | 1.16 | 1.01 | 1.34 | 0.04 |
| RNA5SP518 | 38.23 | 2.23 | 655.58 | 0.01 |
| LETM2 | 1.80 | 1.19 | 2.74 | 0.01 |
| GLT8D2 | 1.09 | 1.03 | 1.14 | 0.00 |
| MBL2 | 10.21 | 1.54 | 67.60 | 0.02 |
| INHBA | 1.02 | 1.00 | 1.04 | 0.02 |
| AC004825.1 | 16.04 | 1.03 | 250.22 | 0.05 |
| VN1R51P | 2.17 | 1.11 | 4.23 | 0.02 |
| MEMO1P3 | 19.09 | 2.42 | 150.80 | 0.01 |
| OR6M3P | 14.77 | 1.18 | 185.12 | 0.04 |
| AC243562.2 | 1.35 | 1.00 | 1.82 | 0.05 |
| TCTEX1D1 | 4.97 | 2.15 | 11.53 | 0.00 |
| SOAT1 | 1.07 | 1.02 | 1.11 | 0.00 |
| AC068397.2 | 8.32 | 1.21 | 56.99 | 0.03 |
| BEX3 | 1.01 | 1.00 | 1.01 | 0.00 |
| AP001318.2 | 0.84 | 0.73 | 0.97 | 0.02 |
| LRCH2 | 1.29 | 1.06 | 1.58 | 0.01 |
| TRAF2 | 0.94 | 0.89 | 1.00 | 0.04 |
| AP000402.1 | 5.69 | 1.01 | 32.10 | 0.05 |
| FIBIN | 1.05 | 1.01 | 1.09 | 0.01 |
| CEP85 | 0.89 | 0.80 | 1.00 | 0.04 |
| CRB3 | 0.97 | 0.95 | 0.99 | 0.01 |
| GPRIN1 | 0.93 | 0.87 | 1.00 | 0.04 |
| LINC01714 | 7.36 | 2.27 | 23.90 | 0.00 |
| REXO1 | 0.95 | 0.91 | 1.00 | 0.03 |
| MTA2 | 0.97 | 0.95 | 0.99 | 0.00 |
| AC231532.2 | 3.83 | 1.02 | 14.49 | 0.05 |
| GNAS | 1.00 | 1.00 | 1.00 | 0.01 |
| NID1 | 1.01 | 1.00 | 1.02 | 0.02 |
| EFL1P2 | 18.24 | 1.25 | 265.96 | 0.03 |
| RASSF8 | 1.03 | 1.01 | 1.06 | 0.01 |
| AL135903.1 | 10.92 | 1.79 | 66.71 | 0.01 |
| USF1 | 0.96 | 0.93 | 0.99 | 0.02 |
| KIF2C | 0.97 | 0.94 | 1.00 | 0.04 |
| NUP50 | 0.93 | 0.89 | 0.98 | 0.01 |
| AC063944.2 | 10.80 | 1.10 | 105.96 | 0.04 |
| BX255925.2 | 0.40 | 0.18 | 0.87 | 0.02 |
| TTC29 | 1.71 | 1.04 | 2.83 | 0.04 |
| PPP1R3D | 0.88 | 0.80 | 0.96 | 0.00 |
| AP005380.1 | 10.30 | 2.47 | 43.00 | 0.00 |
| GOLGA8T | 96554.06 | 43.38 | 214886417.00 | 0.00 |
| AC091832.1 | 19252.59 | 196.20 | 1889207.41 | 0.00 |
| MTND2P25 | 27.83 | 1.78 | 435.38 | 0.02 |
| OR10J1 | 344.20 | 9.90 | 11969.12 | 0.00 |
| AP000720.2 | 14.95 | 2.23 | 100.43 | 0.01 |
| AC002480.1 | 3.48 | 1.89 | 6.40 | 0.00 |
| AC018710.1 | 4.30 | 1.22 | 15.15 | 0.02 |
| RNA5SP45 | 50.47 | 5.85 | 435.66 | 0.00 |
| RAB19 | 0.86 | 0.78 | 0.94 | 0.00 |
| AC140125.1 | 2.70 | 1.38 | 5.28 | 0.00 |
| AC026371.1 | 3.30 | 1.03 | 10.54 | 0.04 |
| AC084364.3 | 10.15 | 2.17 | 47.51 | 0.00 |
| UBE3AP2 | 16.66 | 1.44 | 192.24 | 0.02 |
| AC090579.1 | 0.58 | 0.37 | 0.92 | 0.02 |
| PPIH | 0.97 | 0.95 | 1.00 | 0.04 |
| KCNT2 | 2.43 | 1.38 | 4.26 | 0.00 |
| FRMD7 | 1478.65 | 25.31 | 86382.18 | 0.00 |
| AC091953.6 | 45.67 | 3.65 | 571.92 | 0.00 |
| MIR1185-1 | 5.60 | 2.22 | 14.17 | 0.00 |
| GFAP | 6.04 | 1.92 | 19.00 | 0.00 |
| PABPC1P5 | 81.41 | 3.00 | 2211.98 | 0.01 |
| AL590705.3 | 2.04 | 1.22 | 3.40 | 0.01 |
| MTCO3P30 | 60.33 | 3.04 | 1198.40 | 0.01 |
| AC100797.1 | 0.43 | 0.21 | 0.85 | 0.02 |
| AL590666.2 | 0.96 | 0.94 | 0.99 | 0.00 |
| IL6-AS1 | 3.48 | 1.22 | 9.97 | 0.02 |
| CDH11 | 1.04 | 1.01 | 1.07 | 0.00 |
| OR52L2P | 407.30 | 26.89 | 6170.58 | 0.00 |
| LDB2 | 1.12 | 1.05 | 1.19 | 0.00 |
| PLIN2 | 1.02 | 1.00 | 1.04 | 0.03 |
| CTHRC1 | 1.01 | 1.00 | 1.02 | 0.00 |
| HSPB2 | 1.57 | 1.08 | 2.26 | 0.02 |
| AC025252.2 | 10.22 | 1.68 | 62.09 | 0.01 |
| CLDND1 | 1.04 | 1.00 | 1.08 | 0.04 |
| AC006499.5 | 83505.62 | 237.60 | 29348401.34 | 0.00 |
| ZNF736P8Y | 20418.13 | 10.36 | 40230806.33 | 0.01 |
| AC108734.3 | 7.38 | 2.03 | 26.81 | 0.00 |
| AL772202.1 | 6.09 | 1.34 | 27.71 | 0.02 |
| AC026468.1 | 0.12 | 0.03 | 0.59 | 0.01 |
| AC104260.1 | 4.11 | 1.02 | 16.49 | 0.05 |
| OR1C1 | 32.17 | 1.79 | 579.51 | 0.02 |
| AC113146.1 | 7.47 | 1.63 | 34.29 | 0.01 |
| REPIN1 | 0.99 | 0.98 | 1.00 | 0.01 |
| AP002414.2 | 19.20 | 3.85 | 95.68 | 0.00 |
| OR5L2 | 13.92 | 1.70 | 113.72 | 0.01 |
| LINC02258 | 9.86 | 1.61 | 60.57 | 0.01 |
| HAUS5 | 0.87 | 0.79 | 0.97 | 0.01 |
| NCKAP5-AS2 | 1.80 | 1.26 | 2.56 | 0.00 |
| RPSAP57 | 28.79 | 4.40 | 188.52 | 0.00 |
| AC133485.1 | 45.58 | 3.23 | 642.75 | 0.00 |
| AC055758.2 | 15.92 | 1.18 | 215.64 | 0.04 |
| RNA5SP508 | 6.39 | 1.99 | 20.52 | 0.00 |
| AC026353.1 | 25.53 | 2.91 | 223.86 | 0.00 |
| CGB8 | 1.12 | 1.06 | 1.19 | 0.00 |
| DDO | 1.23 | 1.04 | 1.47 | 0.02 |
| AATK | 0.95 | 0.91 | 1.00 | 0.03 |
| ABCA8 | 1.12 | 1.03 | 1.22 | 0.01 |
| AC010928.3 | 141.51 | 5.87 | 3412.54 | 0.00 |
| OGN | 1.01 | 1.00 | 1.01 | 0.05 |
| AP005597.2 | 32372.81 | 42.94 | 24403624.23 | 0.00 |
| HSD11B1-AS1 | 1.25 | 1.01 | 1.56 | 0.04 |
| AC090802.1 | 12.88 | 1.70 | 97.42 | 0.01 |
| AP000873.4 | 0.46 | 0.24 | 0.89 | 0.02 |
| LPAR5 | 0.95 | 0.91 | 1.00 | 0.03 |
| AC103808.2 | 9.67 | 1.60 | 58.34 | 0.01 |
| FLT4 | 1.18 | 1.04 | 1.35 | 0.01 |
| AC090825.1 | 1.74 | 1.19 | 2.56 | 0.00 |
| MIR32 | 1.45 | 1.09 | 1.92 | 0.01 |
| AC091826.3 | 3.91 | 1.30 | 11.75 | 0.02 |
| DPP9 | 0.95 | 0.91 | 0.99 | 0.02 |
| LEPROT | 1.05 | 1.02 | 1.08 | 0.00 |
| AL162400.2 | 16.96 | 2.69 | 106.88 | 0.00 |
| FAM98C | 0.88 | 0.79 | 0.99 | 0.03 |
| FBXO6 | 0.97 | 0.94 | 0.99 | 0.02 |
| RNU6-308P | 4.81 | 1.23 | 18.83 | 0.02 |
| FAM216B | 1.69 | 1.06 | 2.69 | 0.03 |
| CST6 | 1.01 | 1.00 | 1.01 | 0.00 |
| LINC01374 | 2.30 | 1.14 | 4.65 | 0.02 |
| AL603832.3 | 0.00 | 0.00 | 0.08 | 0.01 |
| ADAM18 | 11.47 | 1.23 | 106.54 | 0.03 |
| PWP2 | 1.66 | 1.15 | 2.41 | 0.01 |
| AC090136.3 | 18.95 | 1.49 | 240.92 | 0.02 |
| CCDC51 | 0.92 | 0.87 | 0.99 | 0.02 |
| CHERP | 0.96 | 0.93 | 1.00 | 0.04 |
| AC107208.1 | 5.16 | 1.53 | 17.40 | 0.01 |
| AP005597.1 | 184.14 | 8.23 | 4122.45 | 0.00 |
| AC005951.1 | 53.70 | 2.85 | 1010.95 | 0.01 |
| HNRNPK | 0.99 | 0.98 | 1.00 | 0.02 |
| AL358452.1 | 15.56 | 1.96 | 123.21 | 0.01 |
| AP3D1 | 0.97 | 0.95 | 1.00 | 0.03 |
| MAGI2-AS3 | 1.21 | 1.02 | 1.43 | 0.03 |
| TRIM6 | 1.40 | 1.01 | 1.93 | 0.04 |
| LINC01878 | 6.48 | 1.27 | 33.04 | 0.02 |
| MIR6507 | 1.70 | 1.02 | 2.83 | 0.04 |
| GTF2IP1 | 3.17 | 1.29 | 7.77 | 0.01 |
| AL158058.1 | 10.82 | 1.10 | 106.18 | 0.04 |
| RNU1-15P | 2.41 | 1.11 | 5.26 | 0.03 |
| MIR5580 | 0.33 | 0.11 | 0.94 | 0.04 |
| SWSAP1 | 0.61 | 0.42 | 0.88 | 0.01 |
| OR51G2 | 31.85 | 3.35 | 302.62 | 0.00 |
| TRAJ56 | 1.80 | 1.10 | 2.94 | 0.02 |
| LINC02752 | 13.26 | 1.86 | 94.26 | 0.01 |
| ATP6V1G1P7 | 4.39 | 1.04 | 18.64 | 0.04 |
| RNU6-555P | 1164.71 | 6.32 | 214523.99 | 0.01 |
| AC092106.2 | 15.37 | 1.82 | 129.90 | 0.01 |
| AC103925.1 | 8.97 | 1.41 | 56.91 | 0.02 |
| SVEP1 | 1.07 | 1.01 | 1.13 | 0.02 |
| FEN1 | 0.97 | 0.95 | 0.99 | 0.01 |
| AC037198.1 | 1.24 | 1.06 | 1.45 | 0.01 |
| PPDPFL | 2.09 | 1.31 | 3.33 | 0.00 |
| NR2F1-AS1 | 1.34 | 1.01 | 1.77 | 0.04 |
| MIR1258 | 2.52 | 1.21 | 5.23 | 0.01 |
| AC118470.1 | 331.84 | 8.27 | 13319.33 | 0.00 |
| AC027702.1 | 2.35 | 1.01 | 5.51 | 0.05 |
| RPS10P7 | 0.74 | 0.58 | 0.95 | 0.02 |
| POLRMT | 0.95 | 0.92 | 0.98 | 0.00 |
| AC026704.1 | 0.59 | 0.37 | 0.92 | 0.02 |
| GPX3 | 1.00 | 1.00 | 1.01 | 0.02 |
| FTLP17 | 2.14 | 1.07 | 4.28 | 0.03 |
| RN7SKP144 | 22.63 | 3.71 | 138.01 | 0.00 |
| AC093627.7 | 1.77 | 1.21 | 2.58 | 0.00 |
| C8orf48 | 2.87 | 1.39 | 5.93 | 0.00 |
| TAF6L | 0.85 | 0.75 | 0.97 | 0.02 |
| AC113331.1 | 547.56 | 12.56 | 23878.25 | 0.00 |
| RDM1P4 | 1786.22 | 36.28 | 87941.91 | 0.00 |
| SLC2A3 | 1.02 | 1.00 | 1.03 | 0.02 |
| OR4C49P | 51.98 | 3.01 | 898.77 | 0.01 |
| MIR6833 | 231.70 | 2.96 | 18152.69 | 0.01 |
| CLUHP4 | 21.21 | 1.68 | 268.32 | 0.02 |
| ACSS3 | 1.48 | 1.18 | 1.86 | 0.00 |
| AC010280.2 | 3.17 | 1.51 | 6.64 | 0.00 |
| AC002542.5 | 12.34 | 2.02 | 75.45 | 0.01 |
| GSTA7P | 3.87 | 1.43 | 10.51 | 0.01 |
| LINC01303 | 3.41 | 1.12 | 10.37 | 0.03 |
| AC135050.2 | 6014.37 | 10.14 | 3566728.08 | 0.01 |
| ITSN1 | 1.22 | 1.00 | 1.48 | 0.05 |
| EPB41L4B | 0.94 | 0.90 | 0.99 | 0.02 |
| AC012414.5 | 82823.61 | 2.86 | 2398595973.00 | 0.03 |
| POT1-AS1 | 3.48 | 1.07 | 11.38 | 0.04 |
| LINC00482 | 1.34 | 1.02 | 1.76 | 0.04 |
| AL022316.1 | 0.70 | 0.54 | 0.89 | 0.00 |
| AC010266.1 | 42.71 | 4.04 | 451.12 | 0.00 |
| AC231760.1 | 33.78 | 1.93 | 591.52 | 0.02 |
| PLCB1-IT1 | 3.56 | 1.29 | 9.85 | 0.01 |
| CDC34 | 0.98 | 0.96 | 1.00 | 0.03 |
| AC105383.1 | 20.10 | 2.00 | 202.44 | 0.01 |
| AC023827.1 | 22.06 | 1.75 | 278.43 | 0.02 |
| AP001506.1 | 4.91 | 1.07 | 22.50 | 0.04 |
| RPL32P35 | 297.00 | 5.00 | 17632.24 | 0.01 |
| PLGRKT | 0.97 | 0.94 | 0.99 | 0.02 |
| CGB2 | 1.56 | 1.02 | 2.36 | 0.04 |
| PCNX3 | 0.97 | 0.94 | 0.99 | 0.01 |
| NBPF22P | 40.58 | 2.84 | 579.50 | 0.01 |
| AP003789.1 | 24.38 | 1.71 | 348.27 | 0.02 |
| MARVELD1 | 1.01 | 1.00 | 1.03 | 0.02 |
| MAGED4B | 1773.31 | 7.95 | 395665.69 | 0.01 |
| AC006963.2 | 4.67 | 1.30 | 16.79 | 0.02 |
| KIF15 | 0.89 | 0.82 | 0.98 | 0.02 |
| MTATP6P23 | 7.71 | 2.15 | 27.62 | 0.00 |
| AL355375.2 | 31.68 | 3.05 | 328.81 | 0.00 |
| AC093664.1 | 9.99 | 1.28 | 78.11 | 0.03 |
| OR51A5P | 6361.31 | 34.60 | 1169683.46 | 0.00 |
| LINC02379 | 6.46 | 1.28 | 32.67 | 0.02 |
| ZNF114P1 | 32.66 | 2.53 | 421.11 | 0.01 |
| RANGAP1 | 0.97 | 0.95 | 0.99 | 0.02 |
| AC117500.5 | 244.50 | 8.95 | 6678.03 | 0.00 |
| AP001189.1 | 1.84 | 1.05 | 3.23 | 0.03 |
| SPC25 | 0.94 | 0.88 | 1.00 | 0.04 |
| NRP1 | 1.06 | 1.04 | 1.09 | 0.00 |
| RPL7P16 | 1.84 | 1.06 | 3.18 | 0.03 |
| MTCO2P6 | 4.88 | 1.03 | 23.21 | 0.05 |
| CYP4A22-AS1 | 0.56 | 0.34 | 0.92 | 0.02 |
| EFNA4 | 0.97 | 0.94 | 0.99 | 0.02 |
| PEX26 | 0.83 | 0.71 | 0.96 | 0.01 |
| NGF | 1.41 | 1.14 | 1.73 | 0.00 |
| MYO5C | 0.95 | 0.91 | 0.99 | 0.03 |
| NUAK1 | 1.09 | 1.00 | 1.18 | 0.04 |
| TNFAIP2 | 0.99 | 0.99 | 1.00 | 0.04 |
| F5 | 1.01 | 1.00 | 1.02 | 0.04 |
| RN7SL283P | 61.91 | 6.05 | 633.91 | 0.00 |
| AL139147.1 | 3.76 | 1.50 | 9.45 | 0.00 |
| MKNK2 | 0.98 | 0.96 | 0.99 | 0.00 |
| AC012501.3 | 1.29 | 1.06 | 1.58 | 0.01 |
| LINC02330 | 62.95 | 3.44 | 1150.44 | 0.01 |
| GPR1 | 1.59 | 1.10 | 2.29 | 0.01 |
| AC003985.1 | 5.41 | 1.72 | 17.01 | 0.00 |
| ZDHHC19 | 3.90 | 1.26 | 12.05 | 0.02 |
| AC100807.1 | 39.71 | 3.67 | 429.19 | 0.00 |
| AC239600.2 | 10.83 | 1.43 | 81.89 | 0.02 |
| PRR5 | 0.89 | 0.81 | 0.97 | 0.01 |
| AL137784.1 | 2.71 | 1.45 | 5.04 | 0.00 |
| AL008638.2 | 66.14 | 4.16 | 1051.29 | 0.00 |
| DNM1P47 | 6441732.17 | 460.48 | 90114890503.00 | 0.00 |
| CNTN1 | 1.04 | 1.00 | 1.07 | 0.03 |
| PDSS1 | 0.96 | 0.93 | 1.00 | 0.05 |
| CLIP4 | 1.12 | 1.03 | 1.22 | 0.01 |
| UPK1B | 1.02 | 1.01 | 1.03 | 0.00 |
| AL355390.1 | 51.45 | 2.10 | 1260.86 | 0.02 |
| AC007557.4 | 1209.76 | 46.95 | 31170.98 | 0.00 |
| AL590226.1 | 2.06 | 1.07 | 3.98 | 0.03 |
| COMMD10 | 1.07 | 1.01 | 1.13 | 0.02 |
| AC090287.1 | 9.82 | 2.02 | 47.84 | 0.00 |
| LINC02011 | 69.15 | 1.46 | 3281.69 | 0.03 |
| FEM1A | 0.32 | 0.14 | 0.70 | 0.00 |
| AL021154.1 | 0.34 | 0.16 | 0.74 | 0.01 |
| ESPL1 | 0.93 | 0.87 | 0.99 | 0.03 |
| RNA5SP117 | 406.93 | 7.71 | 21489.35 | 0.00 |
| AC084759.3 | 4.34 | 1.18 | 16.02 | 0.03 |
| AF254982.1 | 14.92 | 2.69 | 82.77 | 0.00 |
| AL034399.2 | 14.29 | 2.67 | 76.54 | 0.00 |
| AL133247.1 | 5.19 | 1.26 | 21.32 | 0.02 |
| RNA5SP413 | 2.97 | 1.28 | 6.89 | 0.01 |
| ZNF331 | 1.08 | 1.01 | 1.16 | 0.02 |
| MAGEA7P | 9.04 | 1.07 | 76.30 | 0.04 |
| FAP | 1.07 | 1.01 | 1.14 | 0.02 |
| HNRNPM | 0.97 | 0.95 | 0.99 | 0.01 |
| AP001541.1 | 30.72 | 2.57 | 366.85 | 0.01 |
| AL161785.1 | 1.05 | 1.02 | 1.08 | 0.00 |
| AC099552.1 | 29.78 | 3.00 | 295.64 | 0.00 |
| FBXW9 | 0.86 | 0.77 | 0.96 | 0.01 |
| RNU6-1012P | 4.63 | 1.75 | 12.24 | 0.00 |
| PRKCSH | 0.99 | 0.98 | 1.00 | 0.01 |
| REXO1L8P | 85.16 | 1.69 | 4295.55 | 0.03 |
| AL139003.1 | 12.71 | 1.95 | 82.90 | 0.01 |
| AC130289.2 | 1.20 | 1.05 | 1.37 | 0.01 |
| RNVU1-6 | 0.70 | 0.50 | 0.97 | 0.03 |
| LNCPRESS2 | 1784.64 | 42.64 | 74694.88 | 0.00 |
| MTND5P13 | 76.40 | 3.19 | 1830.08 | 0.01 |
| GAPDHP75 | 62.71 | 4.32 | 909.87 | 0.00 |
| SLC6A9 | 0.92 | 0.86 | 0.98 | 0.02 |
| MFSD13A | 0.88 | 0.81 | 0.97 | 0.01 |
| MIR6769B | 3.81 | 1.07 | 13.52 | 0.04 |
| AP007216.2 | 1.83 | 1.31 | 2.57 | 0.00 |
| AL356417.2 | 2.15 | 1.41 | 3.29 | 0.00 |
| BLMH | 1.05 | 1.00 | 1.11 | 0.04 |
| DPH2 | 0.95 | 0.91 | 0.99 | 0.01 |
| TNP1 | 11.26 | 1.99 | 63.84 | 0.01 |
| ZKSCAN7P1 | 76.61 | 3.31 | 1773.98 | 0.01 |
| AC011840.3 | 6.66 | 1.28 | 34.65 | 0.02 |
| CLGN | 1.13 | 1.02 | 1.25 | 0.01 |
| PRKD1 | 1.20 | 1.05 | 1.37 | 0.01 |
| DEPP1 | 1.01 | 1.00 | 1.01 | 0.00 |
| AC022523.2 | 13.52 | 3.07 | 59.58 | 0.00 |
| TMEM120B | 0.70 | 0.53 | 0.93 | 0.01 |
| GRAMD4P3 | 2.33 | 1.09 | 5.00 | 0.03 |
| RAVER1 | 0.96 | 0.93 | 0.99 | 0.01 |
| AL627224.2 | 94.90 | 3.38 | 2667.36 | 0.01 |
| EIF3IP1 | 26.56 | 2.60 | 271.26 | 0.01 |
| RNU6-449P | 8.07 | 2.02 | 32.25 | 0.00 |
| AL592049.1 | 18553.39 | 37.86 | 9091341.76 | 0.00 |
| LINCMD1 | 3.11 | 1.20 | 8.04 | 0.02 |
| AC002350.2 | 0.53 | 0.32 | 0.88 | 0.01 |
| AC093878.1 | 19.47 | 2.44 | 155.51 | 0.01 |
| LINC00519 | 3.05 | 1.52 | 6.13 | 0.00 |
| SHOX2 | 1.35 | 1.05 | 1.72 | 0.02 |
| SNORD114-21 | 1.53 | 1.05 | 2.24 | 0.03 |
| MIR3196 | 1.10 | 1.02 | 1.19 | 0.02 |
| AL121955.1 | 26.61 | 1.73 | 408.61 | 0.02 |
| AC020914.1 | 37.08 | 1.89 | 728.17 | 0.02 |
| TMEM102 | 0.91 | 0.86 | 0.97 | 0.00 |
| RNA5SP422 | 8.62 | 1.73 | 42.88 | 0.01 |
| AC126121.1 | 17.86 | 2.48 | 128.69 | 0.00 |
| RTL8B | 1.05 | 1.01 | 1.08 | 0.01 |
| LINC00412 | 0.22 | 0.07 | 0.66 | 0.01 |
| AC073862.2 | 10.54 | 2.51 | 44.26 | 0.00 |
| AC083964.1 | 1.67 | 1.16 | 2.40 | 0.01 |
| AL357514.1 | 62.84 | 3.47 | 1138.67 | 0.01 |
| AC008667.2 | 11.73 | 1.28 | 107.86 | 0.03 |
| OR10G9 | 727.17 | 6.52 | 81128.77 | 0.01 |
| AL713922.1 | 13923.45 | 39.99 | 4848038.15 | 0.00 |
| MAVS | 0.97 | 0.95 | 1.00 | 0.03 |
| CFH | 1.02 | 1.01 | 1.04 | 0.01 |
| NEK4P1 | 15.11 | 1.26 | 180.95 | 0.03 |
| U91324.1 | 6.73 | 1.05 | 42.97 | 0.04 |
| E2F2 | 0.87 | 0.79 | 0.96 | 0.00 |
| AC105417.1 | 22.93 | 3.62 | 145.10 | 0.00 |
| FAM174C | 0.98 | 0.96 | 1.00 | 0.04 |
| OLFML2A | 1.04 | 1.01 | 1.07 | 0.02 |
| QTRT1 | 0.95 | 0.90 | 0.99 | 0.03 |
| RNA5SP405 | 5.73 | 1.74 | 18.84 | 0.00 |
| HNRNPA1P38 | 4.65 | 1.54 | 14.06 | 0.01 |
| AL359182.1 | 1.42 | 1.12 | 1.81 | 0.00 |
| ALDH3A2 | 0.97 | 0.94 | 0.99 | 0.02 |
| AVPR1A | 1.18 | 1.05 | 1.33 | 0.01 |
| AC017076.1 | 2.98 | 1.31 | 6.79 | 0.01 |
| KCNQ1 | 0.99 | 0.98 | 1.00 | 0.01 |
| MATN3 | 1.07 | 1.03 | 1.10 | 0.00 |
| MORC3 | 1.09 | 1.00 | 1.19 | 0.04 |
| SLC22A17 | 1.06 | 1.01 | 1.10 | 0.01 |
| PYGO1 | 1.19 | 1.03 | 1.38 | 0.02 |
| AC007253.1 | 7.90 | 1.35 | 46.12 | 0.02 |
| CYP4A22 | 8.15 | 1.03 | 64.81 | 0.05 |
| RNA5SP459 | 2.57 | 1.21 | 5.47 | 0.01 |
| NOX4 | 1.64 | 1.24 | 2.17 | 0.00 |
| ZNF101 | 0.81 | 0.69 | 0.96 | 0.01 |
| DNAJB8-AS1 | 110.11 | 2.65 | 4574.38 | 0.01 |
| AC115286.1 | 19.05 | 2.45 | 147.82 | 0.00 |
| LINC02480 | 52.78 | 4.17 | 667.27 | 0.00 |
| LIFR-AS1 | 1.98 | 1.04 | 3.74 | 0.04 |
| TNFRSF10A-AS1 | 0.89 | 0.82 | 0.97 | 0.01 |
| LINC02174 | 5.34 | 1.09 | 26.14 | 0.04 |
| AL442128.1 | 2.06 | 1.16 | 3.66 | 0.01 |
| OR4R2P | 12.26 | 1.69 | 89.24 | 0.01 |
| AC091912.2 | 12.62 | 1.54 | 103.23 | 0.02 |
| VN1R17P | 10.66 | 1.35 | 83.91 | 0.02 |
| TRAV8-7 | 40.15 | 4.83 | 333.60 | 0.00 |
| ABCB5 | 2.90 | 1.09 | 7.70 | 0.03 |
| MIR6792 | 54.13 | 2.76 | 1062.26 | 0.01 |
| AL445493.3 | 2.98 | 1.24 | 7.15 | 0.01 |
| FAM162B | 1.33 | 1.13 | 1.55 | 0.00 |
| KHSRP | 0.99 | 0.98 | 1.00 | 0.03 |
| SOCS2P2 | 3.32 | 1.49 | 7.40 | 0.00 |
| RTL9 | 23.50 | 4.39 | 125.92 | 0.00 |
| FAM241A | 1.23 | 1.06 | 1.43 | 0.01 |
| AC004448.1 | 190.10 | 2.53 | 14255.87 | 0.02 |
| PCNPP3 | 4.32 | 1.35 | 13.75 | 0.01 |
| KRTAP21-1 | 9.41 | 1.65 | 53.62 | 0.01 |
| SNORD115-24 | 3.82 | 1.33 | 11.01 | 0.01 |
| AC013429.2 | 2.95 | 1.51 | 5.75 | 0.00 |
| FRG1 | 0.95 | 0.91 | 0.99 | 0.03 |
| MAP4K4 | 1.02 | 1.00 | 1.04 | 0.04 |
| RCCD1 | 0.89 | 0.80 | 0.99 | 0.03 |
| ZEB2 | 1.11 | 1.01 | 1.22 | 0.03 |
| CYP4F30P | 4.92 | 1.42 | 17.06 | 0.01 |
| DENND1C | 0.93 | 0.87 | 1.00 | 0.04 |
| RBM15 | 0.85 | 0.76 | 0.95 | 0.00 |
| CHCHD10 | 1.00 | 0.99 | 1.00 | 0.04 |
| ABCA9 | 1.29 | 1.06 | 1.57 | 0.01 |
| DNAAF3 | 0.68 | 0.49 | 0.93 | 0.02 |
| TNFAIP8L3 | 1.04 | 1.00 | 1.07 | 0.03 |
| AC020634.1 | 21.90 | 3.81 | 125.77 | 0.00 |
| ADH5P2 | 30.21 | 5.03 | 181.47 | 0.00 |
| AC107399.1 | 5.03 | 1.26 | 20.14 | 0.02 |
| AP000695.2 | 1.28 | 1.10 | 1.48 | 0.00 |
| SAC3D1 | 0.96 | 0.92 | 1.00 | 0.03 |
| GMPPA | 0.95 | 0.91 | 0.99 | 0.02 |
| AC012593.2 | 2.54 | 1.42 | 4.55 | 0.00 |
| RANP9 | 6.09 | 1.21 | 30.68 | 0.03 |
| POSTN | 1.00 | 1.00 | 1.01 | 0.00 |
| RNU6-1312P | 247.85 | 10.80 | 5687.34 | 0.00 |
| RNU7-51P | 5.92 | 1.40 | 25.05 | 0.02 |
| AC092138.1 | 53.45 | 3.07 | 929.68 | 0.01 |
| MIR548AB | 2.35 | 1.23 | 4.51 | 0.01 |
| RPS6KA4 | 0.98 | 0.95 | 1.00 | 0.02 |
| MIR7159 | 4.66 | 1.40 | 15.56 | 0.01 |
| RGS4 | 1.06 | 1.01 | 1.11 | 0.01 |
| ZBTB7A | 0.97 | 0.96 | 0.99 | 0.00 |
| MYF5 | 49.50 | 1.98 | 1239.12 | 0.02 |
| PKIA | 1.15 | 1.03 | 1.29 | 0.02 |
| AC110995.1 | 2.05 | 1.35 | 3.11 | 0.00 |
| ABCA9-AS1 | 2.65 | 1.53 | 4.59 | 0.00 |
| AL445070.1 | 14.12 | 1.21 | 165.19 | 0.03 |
| RGS1 | 1.01 | 1.00 | 1.02 | 0.00 |
| AC012078.1 | 14939.83 | 31.52 | 7081760.02 | 0.00 |
| PTTG1IP | 1.01 | 1.00 | 1.02 | 0.00 |
| AL008626.1 | 5545.81 | 45.21 | 680269.64 | 0.00 |
| CRYGGP | 2.28 | 1.12 | 4.63 | 0.02 |
| FNDC1 | 1.01 | 1.00 | 1.02 | 0.00 |
| PDLIM1P4 | 3.15 | 1.51 | 6.58 | 0.00 |
| SPATA13-AS1 | 0.36 | 0.14 | 0.94 | 0.04 |
| NDC1 | 0.97 | 0.94 | 1.00 | 0.04 |
| CD248 | 1.01 | 1.00 | 1.02 | 0.04 |
| MIR337 | 3.02 | 1.42 | 6.39 | 0.00 |
| COL5A2 | 1.01 | 1.00 | 1.01 | 0.04 |
| RNU6-289P | 7.98 | 2.48 | 25.76 | 0.00 |
| AC004160.1 | 7.41 | 1.28 | 42.87 | 0.03 |
| RAC1P1 | 50.03 | 1.26 | 1989.57 | 0.04 |
| AC087286.2 | 1.30 | 1.02 | 1.66 | 0.03 |
| TRO | 1.25 | 1.01 | 1.55 | 0.04 |
| AC110288.1 | 37.03 | 2.55 | 537.77 | 0.01 |
| RNU7-27P | 2.74 | 1.04 | 7.24 | 0.04 |
| AL365181.3 | 0.95 | 0.92 | 0.99 | 0.01 |
| PPP1R26 | 0.93 | 0.88 | 0.98 | 0.01 |
| SEPTIN7P5 | 39.11 | 1.38 | 1105.83 | 0.03 |
| ANKRD33 | 1.30 | 1.05 | 1.59 | 0.01 |
| PPIAP80 | 2.17 | 1.00 | 4.70 | 0.05 |
| TNK1 | 0.93 | 0.86 | 1.00 | 0.05 |
| CCNT2-AS1 | 1.65 | 1.06 | 2.55 | 0.03 |
| RNU6-205P | 2.97 | 1.05 | 8.45 | 0.04 |
| LINC02789 | 619.90 | 4.85 | 79172.50 | 0.01 |
| AC078843.1 | 2.52 | 1.22 | 5.19 | 0.01 |
| EZH2 | 0.94 | 0.89 | 1.00 | 0.04 |
| AP002892.2 | 10.21 | 1.01 | 102.94 | 0.05 |
| NDOR1 | 0.93 | 0.89 | 0.98 | 0.01 |
| ANAPC2 | 0.92 | 0.86 | 0.99 | 0.02 |
| RNA5SP416 | 3.91 | 1.27 | 12.06 | 0.02 |
| AC090503.2 | 7.64 | 1.93 | 30.23 | 0.00 |
| VGLL3 | 1.03 | 1.00 | 1.06 | 0.03 |
| RNU6-475P | 40.23 | 4.61 | 351.25 | 0.00 |
| RPS14P4 | 0.75 | 0.58 | 0.96 | 0.02 |
| THSD7A | 1.38 | 1.13 | 1.68 | 0.00 |
| RN7SL869P | 6.54 | 1.93 | 22.14 | 0.00 |
| AC092384.1 | 4.25 | 2.01 | 8.98 | 0.00 |
| AC114689.2 | 21.10 | 2.30 | 193.44 | 0.01 |
| AL357312.1 | 22.57 | 1.47 | 345.60 | 0.03 |
| GPR150 | 2.31 | 1.02 | 5.22 | 0.04 |
| LINC02559 | 35.61 | 1.67 | 759.35 | 0.02 |
| AL358944.2 | 7.50 | 1.95 | 28.90 | 0.00 |
| LINC01614 | 1.12 | 1.05 | 1.19 | 0.00 |
| LINC00919 | 797.23 | 20.52 | 30980.28 | 0.00 |
| AC138360.1 | 5.24 | 1.42 | 19.35 | 0.01 |
| GLP2R | 1.33 | 1.04 | 1.70 | 0.02 |
| LINC02272 | 10.92 | 1.38 | 86.21 | 0.02 |
| SELE | 1.06 | 1.02 | 1.12 | 0.01 |
| TSPO | 1.00 | 0.99 | 1.00 | 0.02 |
| ECSIT | 0.97 | 0.94 | 1.00 | 0.04 |
| SNORD115-27 | 18.26 | 1.16 | 287.48 | 0.04 |
| MSI2 | 0.88 | 0.81 | 0.96 | 0.00 |
| KCNS2 | 5.13 | 1.73 | 15.20 | 0.00 |
| THSD7B | 1.35 | 1.03 | 1.78 | 0.03 |
| AC021127.1 | 8.14 | 1.44 | 46.02 | 0.02 |
| AC106785.1 | 281.02 | 6.68 | 11823.32 | 0.00 |
| AC011824.3 | 47.17 | 1.70 | 1307.28 | 0.02 |
| RBMS3 | 1.16 | 1.06 | 1.27 | 0.00 |
| KEAP1 | 0.96 | 0.94 | 0.99 | 0.01 |
| AC091057.2 | 0.84 | 0.74 | 0.95 | 0.01 |
| RPL5P32 | 4.99 | 1.66 | 15.02 | 0.00 |
| ADRA1B | 1.03 | 1.00 | 1.06 | 0.05 |
| AC107058.1 | 81.91 | 2.43 | 2764.40 | 0.01 |
| MAPK10 | 1.44 | 1.11 | 1.86 | 0.01 |
| MIR8084 | 5.94 | 1.80 | 19.62 | 0.00 |
| AC139491.3 | 2.12 | 1.04 | 4.34 | 0.04 |
| PGAP2 | 0.91 | 0.85 | 0.98 | 0.01 |
| SGCE | 1.03 | 1.01 | 1.04 | 0.00 |
| DSTNP5 | 11.58 | 1.40 | 95.57 | 0.02 |
| TGFBR1 | 1.04 | 1.01 | 1.06 | 0.01 |
| NUDT10 | 1.66 | 1.21 | 2.29 | 0.00 |
| RNU6-629P | 1.86 | 1.03 | 3.36 | 0.04 |
| RNU6-1258P | 2.55 | 1.03 | 6.33 | 0.04 |
| CYP19A1 | 2.03 | 1.42 | 2.91 | 0.00 |
| PACRGL | 0.73 | 0.58 | 0.93 | 0.01 |
| AL807761.1 | 19.85 | 2.03 | 194.23 | 0.01 |
| AL592064.1 | 4.63 | 1.22 | 17.58 | 0.02 |
| HP | 1.03 | 1.00 | 1.05 | 0.02 |
| AC018892.2 | 30.77 | 1.33 | 712.29 | 0.03 |
| PIAS4 | 0.93 | 0.87 | 0.99 | 0.02 |
| AC021106.3 | 0.78 | 0.65 | 0.93 | 0.01 |
| IPO5P1 | 0.81 | 0.68 | 0.97 | 0.02 |
| CALML4 | 0.92 | 0.85 | 0.99 | 0.02 |
| MIR6893 | 0.02 | 0.00 | 0.80 | 0.04 |
| EGFLAM | 1.33 | 1.12 | 1.58 | 0.00 |
| KRTAP20-3 | 5.84 | 1.05 | 32.34 | 0.04 |
| AL136309.2 | 28.82 | 3.25 | 255.65 | 0.00 |
| SPRY1 | 1.03 | 1.01 | 1.05 | 0.00 |
| GPR162 | 1.55 | 1.07 | 2.26 | 0.02 |
| SREBF2-AS1 | 0.76 | 0.59 | 0.97 | 0.03 |
| AC005297.3 | 481.31 | 10.63 | 21795.56 | 0.00 |
| SPARC | 1.00 | 1.00 | 1.00 | 0.00 |
| LINC02337 | 6.90 | 1.23 | 38.71 | 0.03 |
| SLC25A15 | 0.91 | 0.85 | 0.98 | 0.01 |
| NPDC1 | 0.99 | 0.98 | 1.00 | 0.03 |
| CAST | 1.03 | 1.01 | 1.05 | 0.01 |
| BASP1 | 1.01 | 1.00 | 1.02 | 0.02 |
| CHAF1A | 0.91 | 0.86 | 0.96 | 0.00 |
| AC007992.1 | 40.09 | 2.38 | 674.11 | 0.01 |
| RNU1-142P | 3.56 | 1.15 | 11.03 | 0.03 |
| KLF5 | 1.00 | 0.99 | 1.00 | 0.02 |
| SLCO2A1 | 1.03 | 1.01 | 1.05 | 0.00 |
| RNU4-55P | 20.05 | 3.05 | 131.82 | 0.00 |
| SMC4P1 | 10.85 | 1.31 | 90.17 | 0.03 |
| TRMT1 | 0.93 | 0.88 | 0.99 | 0.01 |
| TBC1D31 | 0.76 | 0.60 | 0.97 | 0.03 |
| PGAM1P12 | 25.89 | 2.71 | 247.66 | 0.00 |
| ANKRD53 | 1.69 | 1.09 | 2.63 | 0.02 |
| RN7SKP256 | 2.36 | 1.00 | 5.54 | 0.05 |
| IFNA14 | 147.05 | 2.92 | 7412.19 | 0.01 |
| AJ239321.1 | 26.64 | 2.01 | 352.43 | 0.01 |
| MID2 | 1.07 | 1.00 | 1.14 | 0.04 |
| BX470209.1 | 56.21 | 5.37 | 588.42 | 0.00 |
| LOX | 1.03 | 1.01 | 1.04 | 0.00 |
| AC139365.1 | 2.90 | 1.17 | 7.21 | 0.02 |
| FUNDC2P3 | 22.57 | 2.60 | 196.33 | 0.00 |
| CYTL1 | 1.13 | 1.06 | 1.21 | 0.00 |
| PRTG | 1.42 | 1.01 | 1.98 | 0.04 |
| SMPD3 | 0.97 | 0.94 | 0.99 | 0.01 |
| SP6 | 0.92 | 0.87 | 0.98 | 0.00 |
| CHRFAM7A | 3.21 | 1.20 | 8.56 | 0.02 |
| AC147055.2 | 104.68 | 18.05 | 607.11 | 0.00 |
| SLC24A2 | 4.21 | 1.33 | 13.30 | 0.01 |
| STON2 | 1.18 | 1.02 | 1.37 | 0.03 |
| PHLDB3 | 1.11 | 1.01 | 1.22 | 0.04 |
| AC104819.2 | 0.40 | 0.19 | 0.87 | 0.02 |
| AC104985.2 | 6.71 | 1.07 | 41.98 | 0.04 |
| AC078880.1 | 14.15 | 1.23 | 163.55 | 0.03 |
| RPL18AP14 | 40.07 | 2.37 | 676.21 | 0.01 |
| AP001922.2 | 6.93 | 1.14 | 41.97 | 0.04 |
| ORC1 | 0.92 | 0.85 | 0.99 | 0.03 |
| MTND1P20 | 88.08 | 9.04 | 857.87 | 0.00 |
| AC005550.1 | 5.78 | 1.42 | 23.58 | 0.01 |
| MTCYBP20 | 589.67 | 20.20 | 17211.56 | 0.00 |
| RNU6-1230P | 30.74 | 3.94 | 239.72 | 0.00 |
| WDR17 | 1.47 | 1.05 | 2.06 | 0.03 |
| ANO4 | 3.00 | 1.20 | 7.49 | 0.02 |
| AC136777.1 | 60.38 | 10.23 | 356.37 | 0.00 |
| TRAJ51 | 1.69 | 1.20 | 2.37 | 0.00 |
| RFXAP | 0.84 | 0.73 | 0.96 | 0.01 |
| AL358292.1 | 17.27 | 2.11 | 141.32 | 0.01 |
| AC027031.2 | 1.33 | 1.01 | 1.75 | 0.04 |
| OR12D1 | 5662.30 | 23.09 | 1388628.49 | 0.00 |
| MICU3 | 1.33 | 1.06 | 1.67 | 0.01 |
| GPRASP2 | 1.19 | 1.02 | 1.39 | 0.03 |
| AC092142.1 | 1.26 | 1.02 | 1.56 | 0.03 |
| TTC12 | 0.79 | 0.63 | 0.98 | 0.03 |
| GABARAPL2 | 1.05 | 1.02 | 1.09 | 0.00 |
| CDRT8 | 8.71 | 1.22 | 62.23 | 0.03 |
| CNRIP1 | 1.12 | 1.03 | 1.21 | 0.01 |
| AL359924.1 | 37.23 | 2.79 | 497.46 | 0.01 |
| GRB10 | 1.05 | 1.00 | 1.11 | 0.04 |
| RAB34 | 1.02 | 1.01 | 1.04 | 0.00 |
| VPS53 | 0.84 | 0.71 | 1.00 | 0.05 |
| UBE2B | 1.05 | 1.01 | 1.08 | 0.01 |
| AC107464.3 | 1.55 | 1.07 | 2.26 | 0.02 |
| AL049758.1 | 0.26 | 0.07 | 0.92 | 0.04 |
| GADD45B | 1.01 | 1.00 | 1.03 | 0.03 |
| AC023347.2 | 21.86 | 1.78 | 267.81 | 0.02 |
| CYB5RL | 0.65 | 0.43 | 0.98 | 0.04 |
| AC010457.1 | 3.08 | 1.76 | 5.41 | 0.00 |
| AC099344.2 | 8.09 | 1.65 | 39.60 | 0.01 |
| BICC1 | 1.07 | 1.02 | 1.13 | 0.01 |
| CTSL3P | 2.52 | 1.11 | 5.73 | 0.03 |
| EBF2 | 1.29 | 1.05 | 1.59 | 0.02 |
| FMO1 | 1.11 | 1.01 | 1.22 | 0.03 |
| SNORD115-31 | 2.07 | 1.06 | 4.04 | 0.03 |
| AL021392.1 | 2.19 | 1.10 | 4.39 | 0.03 |
| OPLAH | 0.97 | 0.96 | 0.99 | 0.01 |
| TACC1 | 1.02 | 1.00 | 1.03 | 0.03 |
| AC073465.1 | 3.71 | 1.07 | 12.89 | 0.04 |
| GDPGP1 | 0.74 | 0.59 | 0.94 | 0.01 |
| SH3BP1 | 0.95 | 0.92 | 0.98 | 0.00 |
| OR4Q2 | 9269.31 | 1.53 | 56159167.44 | 0.04 |
| FGF7 | 1.04 | 1.01 | 1.08 | 0.01 |
| AC008992.1 | 9.97 | 1.67 | 59.54 | 0.01 |
| CHSY3 | 1.34 | 1.06 | 1.69 | 0.01 |
| SNORD114-6 | 2.31 | 1.22 | 4.37 | 0.01 |
| AC005498.3 | 3.01 | 1.24 | 7.33 | 0.01 |
| AL512357.1 | 5.40 | 1.31 | 22.32 | 0.02 |
| IQCC | 0.68 | 0.51 | 0.92 | 0.01 |
| OR10R3P | 42.08 | 2.36 | 750.95 | 0.01 |
| OR4G6P | 2703.71 | 7.44 | 982179.81 | 0.01 |
| CCN2 | 1.00 | 1.00 | 1.00 | 0.02 |
| CALM2 | 1.01 | 1.00 | 1.02 | 0.01 |
| AC021506.2 | 37.76 | 1.57 | 908.20 | 0.03 |
| AC095350.1 | 11.96 | 1.08 | 131.97 | 0.04 |
| OR10AF1P | 173.61 | 5.53 | 5450.68 | 0.00 |
| IQCA1L | 5.90 | 1.10 | 31.66 | 0.04 |
| AL109653.1 | 11.35 | 1.87 | 68.87 | 0.01 |
| ADAMTS2 | 1.43 | 0.84 | 1.56 | 0.48 |
| BGN | 0.87 | 0.46 | 0.86 | 0.06 |
| BUB1 | 1.57 | 1.05 | 1.87 | 0.05 |
| CDH3 | 0.49 | 0.38 | 1.35 | 0.07 |
| CPEB2 | 1.35 | 0.96 | 1.46 | 0.05 |
| FCGR1B | 0.86 | 0.65 | 1.32 | 0.05 |
| FKBP10 | 1.13 | 1.04 | 1.34 | 0.01 |
| HOXA10 | 0.77 | 0.64 | 0.89 | 0.03 |
| HOXC10 | 0.90 | 0.61 | 0.98 | 0.00 |
| HOXC6 | 1.44 | 1.59 | 1.76 | 0.00 |
| IL13RA2 | 1.26 | 1.00 | 1.50 | 0.04 |
| P3H2 | 1.42 | 1.08 | 1.73 | 0.00 |
| MSR1 | 1.23 | 1.14 | 1.57 | 0.03 |
| NOX4 | 1.25 | 0.89 | 1.77 | 0.05 |
| OLFML2B | 1.42 | 1.13 | 1.66 | 0.03 |
| SALL4 | 0.84 | 0.77 | 0.97 | 0.00 |
| SFRP4 | 0.58 | 0.43 | 0.69 | 0.01 |
| TEAD4 | 1.16 | 1.00 | 1.27 | 0.04 |
| TMEM158 | 0.83 | 0.68 | 0.99 | 0.04 |
| TNFSF4 | 0.83 | 0.46 | 0.97 | 0.00 |
| WISP1 | 0.76 | 0.58 | 1.13 | 0.04 |
| WISP3 | 1.23 | 1.00 | 1.43 | 0.03 |
| SULF1 | 0.84 | 0.69 | 1.24 | 0.05 |
| PMEPA1 | 1.17 | 1.07 | 1.25 | 0.00 |
| CEMIP | 0.91 | 0.72 | 1.01 | 0.00 |
| PMM1 | 1.26 | 1.04 | 1.34 | 0.06 |
| INHBA | 1.38 | 0.99 | 1.77 | 0.04 |
