# Supplementary material for: Identification of ALDH3A2 as a novel prognostic biomarker in gastric adenocarcinoma using integrated bioinformatics analysis
Source: BMC Cancer. 2020 Nov 4;20:1062. doi: 10.1186/s12885-020-07493-x (PMC7640415; doi:10.1186/s12885-020-07493-x)
Supplement: Supplementary file 5 — Additional file 5: Table S2. The 31 genes of prognosis model. [file 12885_2020_7493_MOESM5_ESM.docx]

| **Table S2. The 31 genes of prognosis model** | | | | | |
| --- | --- | --- | --- | --- | --- |
| Gene | coef | HR | HR.95L | HR.95H | pvalue |
| DOK6 | 0.28 | 1.33 | 0.95 | 1.84 | 0.09 |
| ALDH3A2 | -0.33 | 0.72 | 0.58 | 0.89 | 0.00 |
| BDH2 | 0.29 | 1.33 | 1.09 | 1.64 | 0.01 |
| SUGT1 | -0.48 | 0.62 | 0.40 | 0.96 | 0.03 |
| AC107626.1 | 0.23 | 1.26 | 1.01 | 1.56 | 0.04 |
| POF1B | -0.19 | 0.83 | 0.68 | 1.01 | 0.07 |
| ACBD5 | -0.19 | 0.83 | 0.68 | 1.00 | 0.05 |
| GLUD2 | 0.16 | 1.17 | 0.97 | 1.42 | 0.09 |
| CTHRC1 | 0.51 | 1.66 | 1.24 | 2.22 | 0.00 |
| PEX5 | -0.29 | 0.75 | 0.56 | 1.00 | 0.05 |
| MAB21L2 | 0.03 | 1.03 | 1.01 | 1.06 | 0.00 |
| FNDC1 | 0.48 | 1.61 | 1.19 | 2.17 | 0.00 |
| MTPAP | -0.18 | 0.84 | 0.71 | 0.99 | 0.04 |
| VHL | -0.26 | 0.77 | 0.63 | 0.95 | 0.02 |
| NAV3 | 0.29 | 1.34 | 1.11 | 1.62 | 0.00 |
| IGFBP7 | 0.19 | 1.20 | 1.01 | 1.44 | 0.04 |
| LHX6 | 0.29 | 1.34 | 0.97 | 1.85 | 0.08 |
| GRP | 0.36 | 1.44 | 1.11 | 1.87 | 0.01 |
| DCLK1 | 0.29 | 1.34 | 0.93 | 1.94 | 0.12 |
| AP001858.2 | 0.30 | 1.35 | 1.05 | 1.73 | 0.02 |
| GLE1 | 0.33 | 1.40 | 1.16 | 1.68 | 0.00 |
| MCEMP1 | 0.14 | 1.16 | 1.06 | 1.26 | 0.00 |
| ART4 | 0.18 | 1.20 | 1.01 | 1.42 | 0.04 |
| AP001107.6 | -0.03 | 0.97 | 0.94 | 1.00 | 0.09 |
| TRIM25 | -0.56 | 0.57 | 0.41 | 0.78 | 0.00 |
| MRPL4 | -0.31 | 0.74 | 0.53 | 1.03 | 0.08 |
| ANKRD6 | 0.09 | 1.09 | 0.99 | 1.21 | 0.08 |
| KIF11 | -0.28 | 0.76 | 0.59 | 0.98 | 0.03 |
| GMEB1 | -0.35 | 0.71 | 0.53 | 0.94 | 0.02 |
| PDXK | -0.28 | 0.76 | 0.57 | 1.00 | 0.05 |
| APOD | 0.15 | 1.16 | 0.98 | 1.36 | 0.08 |
